# Nanocellulose Paper Semiconductor with a 3D Network Structure and Its Nano–Micro–Macro Trans-Scale Design

**DOI:** 10.1021/acsnano.1c10728

**Published:** 2022-04-26

**Authors:** Hirotaka Koga, Kazuki Nagashima, Koichi Suematsu, Tsunaki Takahashi, Luting Zhu, Daiki Fukushima, Yintong Huang, Ryo Nakagawa, Jiangyang Liu, Kojiro Uetani, Masaya Nogi, Takeshi Yanagida, Yuta Nishina

**Affiliations:** † SANKEN (The Institute of Scientific and Industrial Research), Osaka University, 8-1 Mihogaoka, Ibaraki, Osaka 567-0047, Japan; ‡ Department of Applied Chemistry, Graduate School of Engineering, The University of Tokyo, 7-3-1 Hongo, Bunkyo-ku, Tokyo 113-8656, Japan; § Japan Science and Technology Agency (JST), PRESTO, 4-1-8 Honcho, Kawaguchi, Saitama 332-0012, Japan; ∥ Department of Advanced Materials Science and Engineering, Faculty of Engineering Sciences, Kyushu University, 6-1 Kasuga-Koen, Kasuga, Fukuoka 816-8580, Japan; ⊥ Graduate School of Natural Science and Technology, Okayama University, 3-1-1 Tsushimanaka, Kita-ku, Okayama 700-8530, Japan; # Institute for Materials Chemistry and Engineering, Kyushu University, 6-1 Kasuga-Koen, Kasuga, Fukuoka 816-8580, Japan; ∇ Research Core for Interdisciplinary Sciences, Okayama University, 3-1-1 Tsushimanaka, Kita-ku, Okayama 700-8530, Japan

**Keywords:** nanocellulose, semiconductor, trans-scale structural design, tunable electrical property, paper electronics, customized 3D network structures

## Abstract

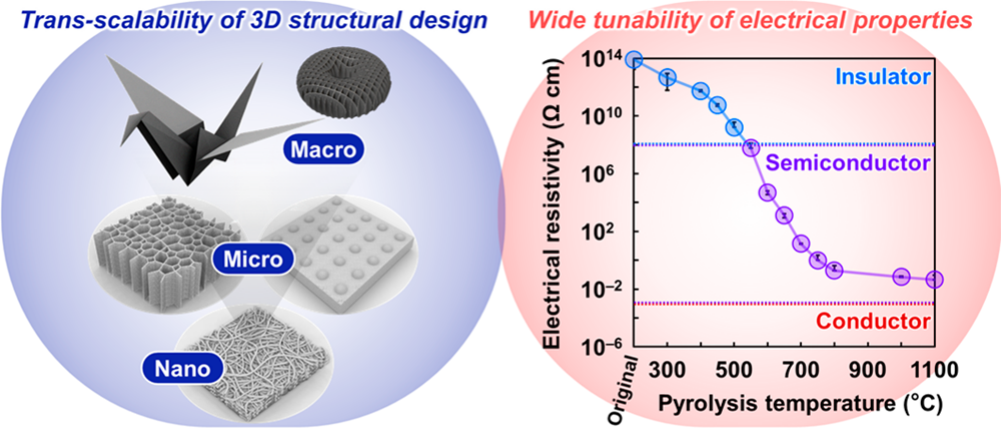

Semiconducting nanomaterials with 3D network structures exhibit various fascinating properties such as electrical conduction, high permeability, and large surface areas, which are beneficial for adsorption, separation, and sensing applications. However, research on these materials is substantially restricted by the limited trans-scalability of their structural design and tunability of electrical conductivity. To overcome this challenge, a pyrolyzed cellulose nanofiber paper (CNP) semiconductor with a 3D network structure is proposed. Its nano–micro–macro trans-scale structural design is achieved by a combination of iodine-mediated morphology-retaining pyrolysis with spatially controlled drying of a cellulose nanofiber dispersion and paper-crafting techniques, such as microembossing, *origami*, and *kirigami*. The electrical conduction of this semiconductor is widely and systematically tuned, *via* the temperature-controlled progressive pyrolysis of CNP, from insulating (10^12^ Ω cm) to quasimetallic (10^–2^ Ω cm), which considerably exceeds that attained in other previously reported nanomaterials with 3D networks. The pyrolyzed CNP semiconductor provides not only the tailorable functionality for applications ranging from water-vapor-selective sensors to enzymatic biofuel cell electrodes but also the designability of macroscopic device configurations for stretchable and wearable applications. This study provides a pathway to realize structurally and functionally designable semiconducting nanomaterials and all-nanocellulose semiconducting technology for diverse electronics.

Customizing 3D network structures ranging from nano- to micro- and macroscales is a promising strategy for fabricating material systems with excellent characteristics and functionalities.^1−3^ The 3D network structures at nano- and microscales exhibit fascinating properties including high permeability and large surface areas, which are crucial for adsorption, separation, sensing, and biomedical applications.^2^ To observe these properties in large-scale objects for macroscopic applications, a macroscale design of the nano–microstructures with a 3D network is required.^3^ Macrostructures with specific geometries can exhibit fascinating mechanical properties such as high compressibility for micropressure sensing^4^ and stretchability for effective light capturing^5^ and heat dissipation.^6^ Thus, the nano–micro–macro trans-scale design of 3D-network-containing structures is important for developing advanced materials with excellent functionality and end-use versatility.

Accordingly, a 3D structural design of semiconducting nanomaterials with tunable electrical conduction properties has been recently reported for advanced electronic applications using synthesis,^7,8^ self-assembly,^9,10^ and additive manufacturing^4,11−16^ methods. The synthetic approach provided 3D metal oxides with nano–microscale porous structures (pore size: 100 nm to several tens of micrometers)^7^ and 3D organic semiconductors with an average pore size of 0.4 nm.^8^ The self-assembly method allowed for the fabrication of 3D Si- or P-doped WO_3_ and MoO_3_ nanowire and 3D CdTe/Au nanoparticle structures with pore sizes of ∼30^9^ and 3–10 nm,^10^ respectively. Additive manufacturing techniques, such as 3D printing, afford 3D micro–macrostructural designs of reduced graphene oxide,^4,11,12^ metal oxides,^13−15^ and intermetallic compounds.^16^ Despite these efforts, the nano–micro–macro trans-scale design of 3D semiconductor structures has been seldom achieved to date. Modification of the electrical conduction properties of 3D semiconductor structures has also been reported; the electrical resistivities of 3D porous organic polymers,^8^ 3D metal–organic frameworks,^17^ and 3D reduced graphene oxides^18,19^ were tuned from 10^8^ to 10^4^, 10^7^ to 10^1^, and 10^2^ to 10^0^ Ω cm, respectively, by impurity doping^8,17^ and control of material density.^18,19^ Tuning of the electrical conduction properties of semiconductors allows for the expansion of their functionality and applicability to diverse electronics. However, the applicability of 3D semiconductor structures has been substantially restricted by the limited tunability of their electrical conduction properties because the electrical resistivities of conventional 3D semiconducting nanomaterials can be controlled to a limited range. Thus, both nano–micro–macro trans-scale designing of 3D semiconductor structures and wide tuning of their electrical properties still remain a challenge.

Herein, 3D-network-structured semiconducting nanomaterials based on wood-derived cellulose nanofiber paper (CNP) with both trans-scalability of structural design and wide tunability of electrical conduction properties are proposed. The CNP containing 3D nanofiber-network structures with a large area of >20 cm in diameter can be constructed using cellulose nanofibers as nano building blocks.^20,21^ CNP also has paperlike mechanical properties such as flexibility and shapability, thereby showing excellent potential for the trans-scalability of structural design. Furthermore, cellulose nanofibers have a high electrical resistance (>10^14^ Ω)^22^ because of the presence of sp^3^-hybridized carbons in cellulose molecules;^23^ therefore, CNP is a promising starting material for the wide tuning of electrical conduction properties. Although high-temperature pyrolysis is an effective method to decrease the electrical resistivity of cellulose nanomaterials,^24^ the original morphology of wood cellulose nanofibers deteriorates upon pyrolysis,^25^ rendering it difficult to design the pyrolyzed CNP structure. Herein, 3D nano–micro–macro trans-scale structural design and electrical resistivity tuning of the pyrolyzed CNP semiconductor is realized using a morphology-retaining and temperature-controlled progressive pyrolysis strategy. The trans-scalability of structural design can provide a pyrolyzed CNP semiconductor with high functionality and designability of macroscopic device configurations, thereby affording stretchable and wearable electronics by integration on a paper substrate. The electrical resistivity of the pyrolyzed CNP semiconductor is tuned from 10^12^ to 10^–2^ Ω cm, expanding its applicability range from water vapor sensors to electrodes of enzymatic biofuel cells for energy generation.

## Results and Discussion

### 3D Nano–Micro–Macro Trans-Scale Structural Design and Morphology-Retaining Pyrolysis of CNP

**Figure 1:**
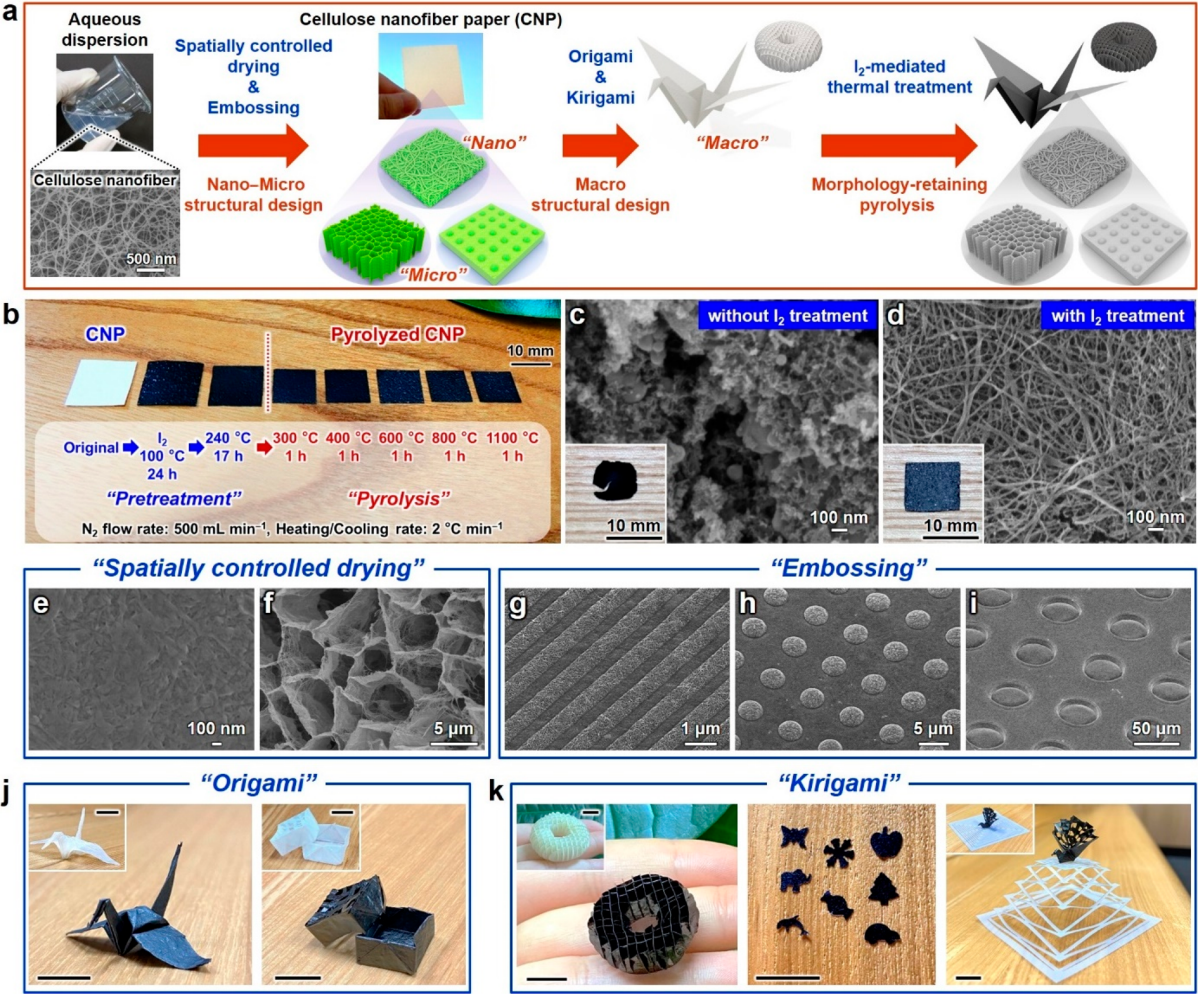
Nano–micro–macro trans-scale crafting and morphology-retaining pyrolysis of cellulose nanofiber paper (CNP). Schematics of (a) the nano–micro–macrostructural design and morphology-retaining pyrolysis of CNP and (b) the pretreatment and pyrolysis of CNP at different temperatures. Optical and field-emission scanning electron microscopy (FE-SEM) images of pyrolyzed CNP (c) without and (d) with I_2_ treatment. FE-SEM images of pyrolyzed CNP prepared using (e and f) spatially controlled drying and (g–i) microembossing. Optical images of the (j) *origami*- and (k) *kirigami*-processed CNPs before and after pyrolysis. White and black papers indicate the original and pyrolyzed CNPs, respectively. Scale bar in (j and k): 10 mm. Pyrolysis temperature: 600 °C (e–k) or 1100 °C (c and d).

A 3D nano–micro–macro trans-scale structure design of a CNP semiconductor was achieved using the workflow shown in Figure 1a. Cellulose nanofibers with a width of 22 ± 8 nm and carboxylate content of 0.08 ± 0.02 mmol g^–1^ were prepared using never-dried pulp (softwood bleached kraft pulp) by employing the aqueous counter collision method (Figure S1).^26^ Starting with the aqueous dispersion of cellulose nanofibers, CNP was first fabricated using various manufacturing techniques and then pyrolyzed to modulate its electrical properties. Simple pyrolysis deteriorates the morphology of wood-derived cellulose nanofibers,^25^ rendering the construction of structurally designed CNP semiconductor difficult because high-temperature pyrolysis of organic materials removes carbon and hydrogen as a hydrocarbon gas, thereby weakening their carbon frameworks.^27^ To overcome this limitation, iodine-mediated pyrolysis was performed. Figure 1b shows I_2_-mediated pyrolysis. The apparent volume and weight of pyrolyzed CNP changed significantly than those of the original CNP (Figure S2a). The bulk density of CNP did not change largely by pyrolysis; the bulk density of the original CNP changed from 0.294 ± 0.009 to 0.266 ± 0.013 g cm^–3^ after pyrolysis at 1100 °C (Figure S2b). The specific surface area of CNP increased from 104 ± 10.9 to 721 ± 39.6 m^2^ g^–1^ with an increase in the pyrolysis temperature (Figure S3). Although pyrolyzed CNP was somewhat brittle, it was freestanding and allowed handling for evaluation and applications. Parts c and d of Figure 1 show the optical and field-emission scanning electron microscopy (FE-SEM) images of the CNP pyrolyzed at 1100 °C without and with I_2_ treatment, respectively. The fractured macrostructure and granular-shaped nanostructure were observed without I_2_ treatment, concomitant with the collapse of the nanofiber structure. In contrast, the nanofiber structure and macrostructure were retained after I_2_ treatment. Notably, only ∼2.8% of the CNP weight was retained without I_2_ treatment but increased up to ∼17% with I_2_ treatment. These results indicated that carbon removal during pyrolysis was successfully suppressed by I_2_ treatment, possibly owing to the preferential formation of HI.^28^ Therefore, I_2_-mediated pyrolysis was employed to realize a 3D nano–micro–macro trans-scale structure design of pyrolyzed CNP.

To design the 3D nano–microstructures of pyrolyzed CNP, spatially controlled drying^29,30^ and microembossing^31^ methods were employed. Parts d–f of Figure 1 show the FE-SEM images of pyrolyzed CNP fabricated by spatially controlled drying. When the aqueous dispersion of cellulose nanofibers was dried, a densely packed nanostructure was obtained due to agglomeration (Figure 1e) because of the capillary force generated by the high surface tension of water (72.14 mN m^–1^ at 25 °C).^32^ Solvent exchange with *tert*-butyl alcohol with a low surface tension (*t*-BuOH, 19.96 mN m^–1^ at 25 °C)^32^ and subsequent freeze-drying afforded porous nanofiber-network structures with pore sizes of <100 nm (Figure 1d), which were consistently observed at the examined pyrolysis temperatures (Figure S4). Freeze-drying of concentrated cellulose nanofibers in aqueous dispersion formed honeycomb-shaped microstructures with pore sizes of several micrometers, which are consistent with the shape of an ice crystal (Figure 1f); the pore sizes of pyrolyzed CNP could be controlled from nano- to microscale. The surface structure of pyrolyzed CNP was further crafted by microembossing (Figure 1g–i). Using patterned imprint molds, line-, convex-, and concave-shaped microstructural arrays of up to tens of micrometers in size were embossed on the pyrolyzed CNP surface. To fabricate the 3D macrostructure of pyrolyzed CNP, *origami* and *kirigami* processings^33^ were employed. Crane- and box-shaped 3D macrostructures of pyrolyzed CNP were obtained *viaorigami* processing (Figure 1j), and a waffle-patterned doughnut structure and various punch arts were fabricated *viakirigami* processing (Figure 1k, left and middle images). By integrating the original and pyrolyzed CNPs, a stretchable 3D macrostructure was also obtained (Figure 1k, right image). While the I_2_ treatment has been previously reported for the nanomorphology-retaining pyrolysis of a helical polyacetylene film,^28^ the present study demonstrated the successful retention of the 3D complex nano–micro–macrostructures of the CNP by I_2_-mediated pyrolysis. Thus, the 3D nano–micro–macro trans-scale design of pyrolyzed CNP, ranging from nanometers to centimeters in terms of the pore size, surface structure, and macroscopic morphology, was achieved through the versatile structural designability of CNP in combination with I_2_-mediated morphology-retaining pyrolysis.

### Tunability of Electrical Properties of Pyrolyzed CNP

**Figure 2:**
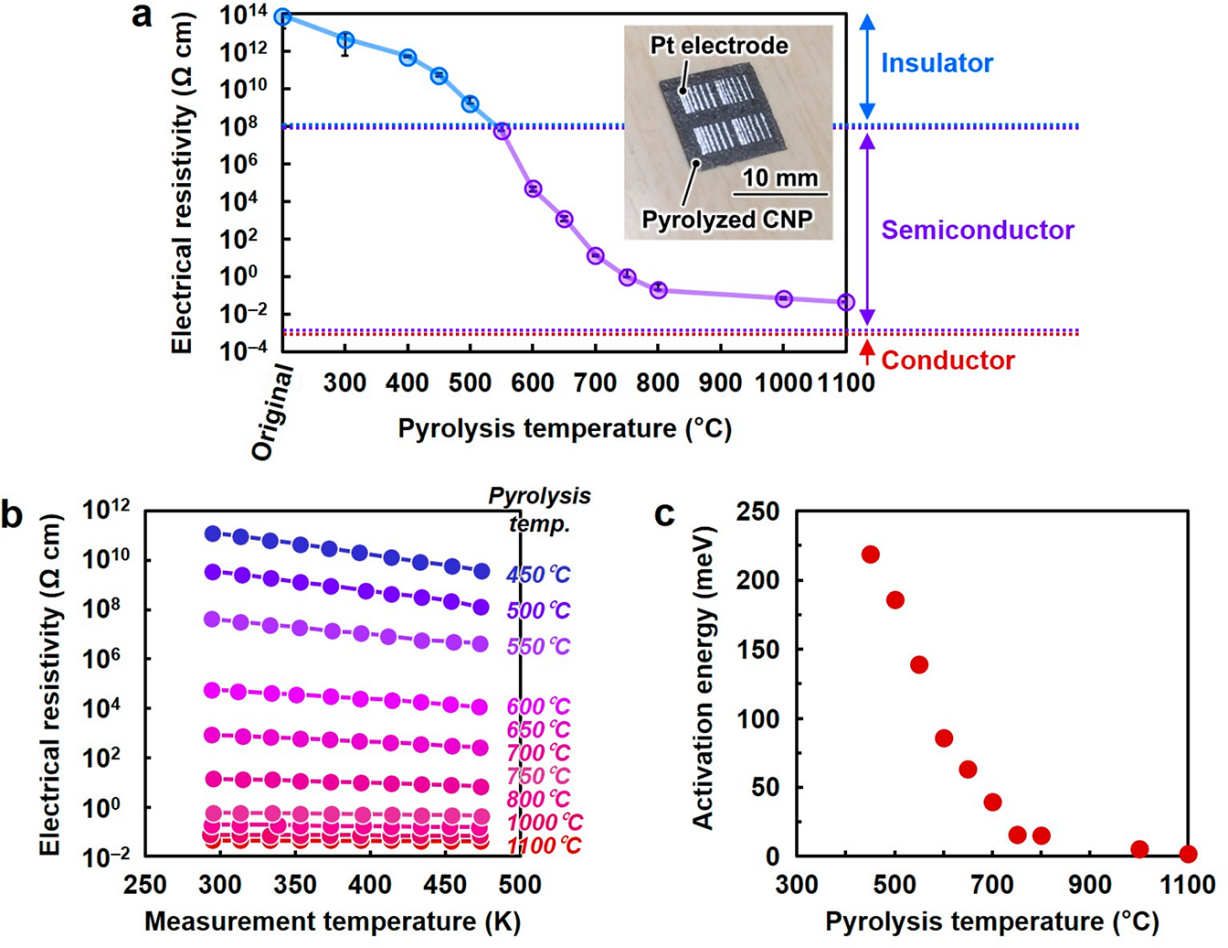
Tunable electrical properties of pyrolyzed cellulose nanofiber paper (CNP). (a) Electrical resistivity of the CNP pyrolyzed at different temperatures. (b) Electrical resistivity of the CNP pyrolyzed at different temperatures versus measurement temperatures. (c) Activation energy of the CNP pyrolyzed at different temperatures. Inset of (a) shows an optical image of a pyrolyzed CNP with Pt electrodes, which was used for the electrical measurement.

To explore the potential of pyrolyzed CNP as a 3D semiconductor, the tunability of its electrical properties was investigated. Figure 2a shows the electrical resistivity of CNP with porous nanostructures pyrolyzed at different temperatures. Inset shows an optical image of a pyrolyzed CNP with Pt electrodes, which was used for the electrical measurement. Pyrolyzed CNP showed ohmic behavior in current–voltage characteristics, indicating the formation of good electrical contacts between pyrolyzed CNP and the electrodes (Figure S5). The original CNP exhibited highly insulating electrical properties with an electrical resistivity of >10^13^ Ω cm owing to the presence of sp^3^-hybridized carbons in cellulose.^23^ The electrical resistivity of CNP was significantly and systematically modulated in the 10^12^–10^–2^ Ω cm range using I_2_-mediated pyrolysis. The degree of tunability of the electrical properties of pyrolyzed CNP was considerably higher than that of the previously reported 3D semiconductor structures^8,17−19,34−36^ as well as doped Si and GaAs, oxidized graphite, reduced graphene oxide, and other semiconducting compounds (Table S1). To further characterize the conduction properties of pyrolyzed CNP, resistivity–temperature (ρ–*T*) curves were evaluated (Figure 2b). At low pyrolysis temperatures, electrical resistivity decreased with increasing measurement temperatures. However, at high pyrolysis temperatures, electrical resistivity was almost constant and independent of the measurement temperature. The former and latter trends are characteristic of semiconductor and quasi-conductor materials, respectively.^37^ The variation in the conduction properties of pyrolyzed CNP was more systematically observed in terms of activation energy, which was calculated from the ρ–*T* curves (Figure 2c). The activation energy decreased from 220 to 1.9 meV with an increase in the pyrolysis temperature from 450 to 1100 °C. The energy bandgap of pyrolyzed CNP was significantly larger than its activation energy (Figures S6–S9). These results implied that the thermal excitation of carriers from or to the midgap states dominated the electrical conduction of pyrolyzed CNP. The carrier concentration of pyrolyzed CNP, determined using Hall effect measurements, increased from ∼10^15^ to 10^20^ cm^–3^ with an increase in the pyrolysis temperature from 650 to 1100 °C (Table 1 and Figure S10). The major carrier types were *n*-rich at 650 °C and *p*-rich at 750, 1000, and 1100 °C, suggesting that electrons and holes contributed to the electrical conduction of the CNPs pyrolyzed at 650 and ≥750 °C, respectively. The carrier mobilities of pyrolyzed CNP were 0.235–2.59 cm^2^ V^–1^ s^–1^, which were not significantly affected by pyrolysis temperature (Table 1). These results suggested that the electrical conduction properties of pyrolyzed CNP could be widely tuned by changing its carrier concentration rather than mobility. Therefore, pyrolyzed CNP exhibited 3D nano–micro–macro trans-scale structural designability and allowed for wide tunability of its electrical properties.

**Table 1:** Carrier Concentration and Carrier Mobility of the CNP Pyrolyzed at Different Temperatures^a^

pyrolysis temperature (°C)	carrier concentration (cm^–3^)	carrier mobility (cm^2^ V^–1^ s^–1^)	carrier type
650	2.89 × 10^15^	2.59	*n*-rich
750	1.60 × 10^19^	0.235	*p*-rich
1000	1.56 × 10^20^	0.614	*p*-rich
1100	1.03 × 10^20^	0.673	*p*-rich

a CNP, cellulose nanofiber paper.

### Molecular Structures of Pyrolyzed CNP

**Figure 3:**
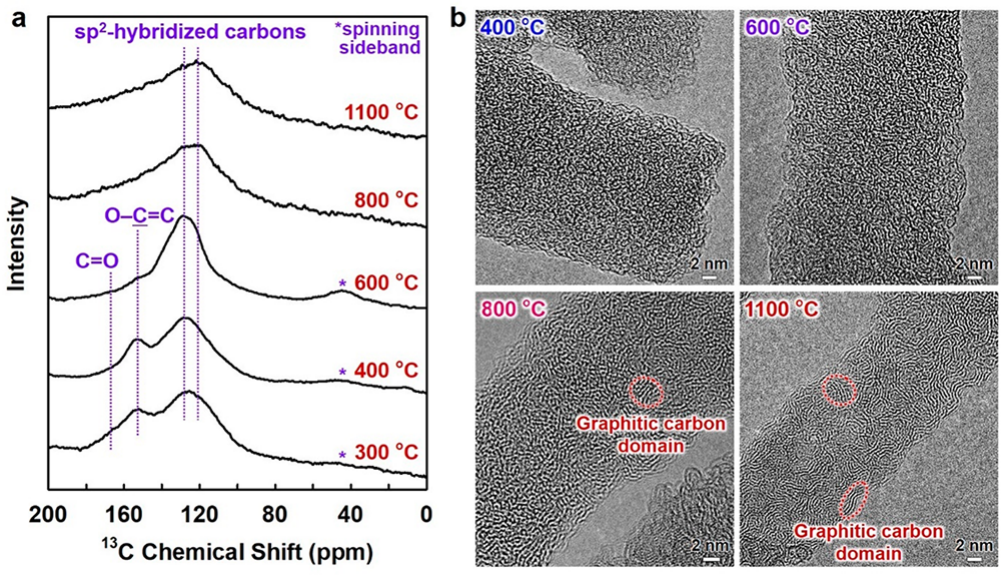
Molecular structures of pyrolyzed cellulose nanofiber. (a) Solid-state ^13^C nuclear magnetic resonance (NMR) spectra and (b) high-resolution transmission electron microscopy (HR-TEM) images for the cellulose nanofiber pyrolyzed at different temperatures.

As the electrical insulating property of the original CNP is derived from sp^3^-hybridized carbon structures present in cellulose,^23^ the wide and systematic variation in the electrical properties of pyrolyzed CNP must be associated with a significant change in its molecular structure. To understand this mechanism, changes in the molecular structure of CNP upon temperature-controlled pyrolysis were analyzed. Solid-state ^13^C nuclear magnetic resonance (NMR) spectra showed that the sp^2^-hybridized carbon domains with oxygen-containing groups (*i*.*e*., disordered regions) such as C=O and O—C=C were formed at low pyrolysis temperatures (Figure 3a). When this temperature was increased to ∼600 °C, the sp^2^-hybridized carbon domains became more prominent with a decrease in the disordered regions of the carbon domains due to the gradual removal of C=O and O—C=C groups. At high pyrolysis temperatures of ≥800 °C, the peak at ∼120 ppm, corresponding to the sp^2^-hybridized carbon, increased in intensity, indicating the formation of more graphite-like carbon structures.^38^ The progressive formation of graphitic carbon domains was observed in the high-resolution transmission electron microscopy (HR-TEM) images (Figure 3b and Figure S11). The results were also confirmed by elemental, Fourier-transform infrared (FT-IR) spectroscopy, Raman spectroscopy, and X-ray diffraction (XRD) analyses (Figures S12 and S13). These results indicated that the progressive growth of sp^2^-hybridized carbon domains gradually narrowed the band gap and thus facilitated interband transitions, leading to the increased carrier concentration and electrical conductivity of pyrolyzed CNP. The major carrier type could be determined by the chemical state of the disordered regions; *n*- and *p*-rich conductions are derived from electron-donating groups such as C=C—O—C=C (furan-like ethers) and electron-withdrawing groups such as C=O, respectively (Figure S14), as reported for reduced graphene oxide.^39^ Hence, progressive pyrolysis of the highly insulating CNP allowed for wide and systematic tunability of electrical properties (Figure S15 and Supporting Note S1).

### Application to Semiconductor Molecular Sensor

**Figure 4:**
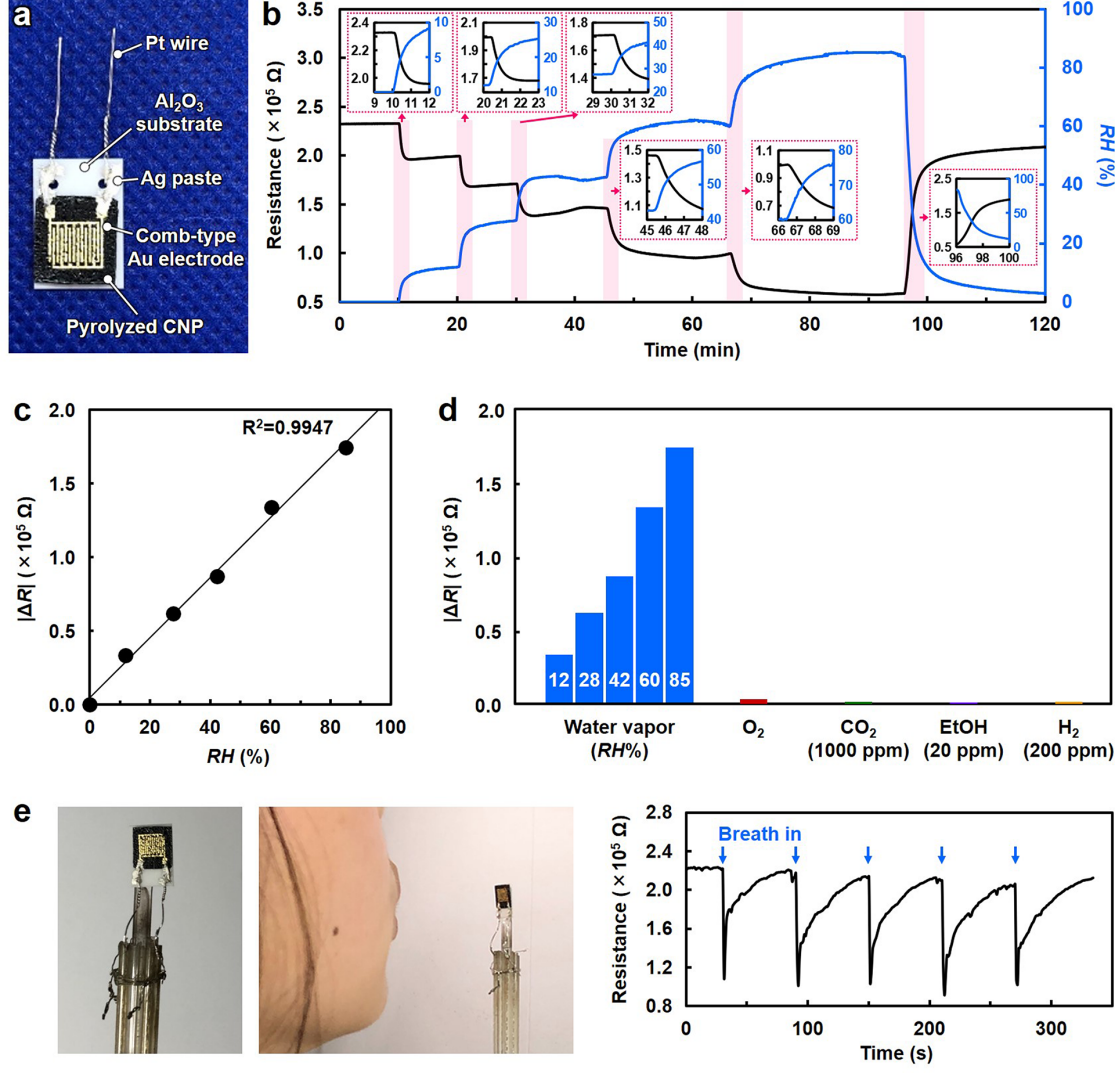
Selective water-vapor sensing performance of pyrolyzed cellulose nanofiber paper (CNP). (a) Optical image of the sensor device consisting of the CNP pyrolyzed at 600 °C and comb-type Au electrodes. (b) Resistance as a function of time upon exposure to water vapor with different relative humidity (RH) values at 30 °C. (c) Change in resistance (|Δ*R*|) as a function of RH. (d) |Δ*R*| upon exposure to water vapor with different RH values, O_2_, CO_2_ (1000 ppm in air), and ethanol (EtOH, 20 ppm in air) at 30 °C, and H_2_ (200 ppm in air) at 50 °C. (e) Water vapor sensing in exhaled human breath (introduction time, 1 s; interval time, 59 s).

3D semiconducting nanomaterials with porous nano–microstructures provide numerous target–receptor interfaces and efficient molecule diffusion in various environments (*e*.*g*., air and solution), which are beneficial for molecular sensing with high sensitivity and fast response/recovery dynamics.^9,40^ Herein, pyrolyzed CNP was applied to a molecular sensing device. The sensor device comprised pyrolyzed CNP and comb-type Au electrodes (Figure 4a). For the device, the CNP pyrolyzed at 600 °C that had a resistivity of the order of 10^4^ Ω cm, C=O, C—O—C, and C—OH groups (Figure S12b), and porous nanostructures (Figure S4) was used as the sensor material. Figure 4b shows the sensor response to water vapor under varying relative humidity (RH) conditions. Measurements were performed at 30 °C in air. With increasing RH, the sensor resistance decreased and varied continuously according to a variation in humidity. Excellent linearity of the sensor resistance to RH was observed (Figure 4c). The cross-sensitivity of the fabricated sensor device to other analyte molecules such as oxygen, carbon dioxide, hydrogen, and ethanol is shown in Figure 4d. Sensor responses to these analyte molecules were sufficiently low, even when their measurement temperature was increased (Figure S16), indicating its high selectivity to water vapor. Furthermore, the device showed a rapid response to water vapor within 1 s and recovery within 40 s with reproducibility, as demonstrated by the detection of human-exhalation-derived water vapor (Figure 4e and Figures S17 and S18). Therefore, the molecular sensing mechanism of pyrolyzed CNP might depend on the polar interaction between water vapor and sensor surface rather than the combustion reaction^41^ because the measurement temperature in this study is sufficiently low. Water vapor, which has a higher polarity than other analyte molecules, preferentially interacts with polar functional groups such as C=O, C—O—C, and C—OH and donates electrons to the midgap states in the pyrolyzed CNP semiconductor, enhancing the *n*-rich electrical conduction (Figure S15b).

**Figure 5:**
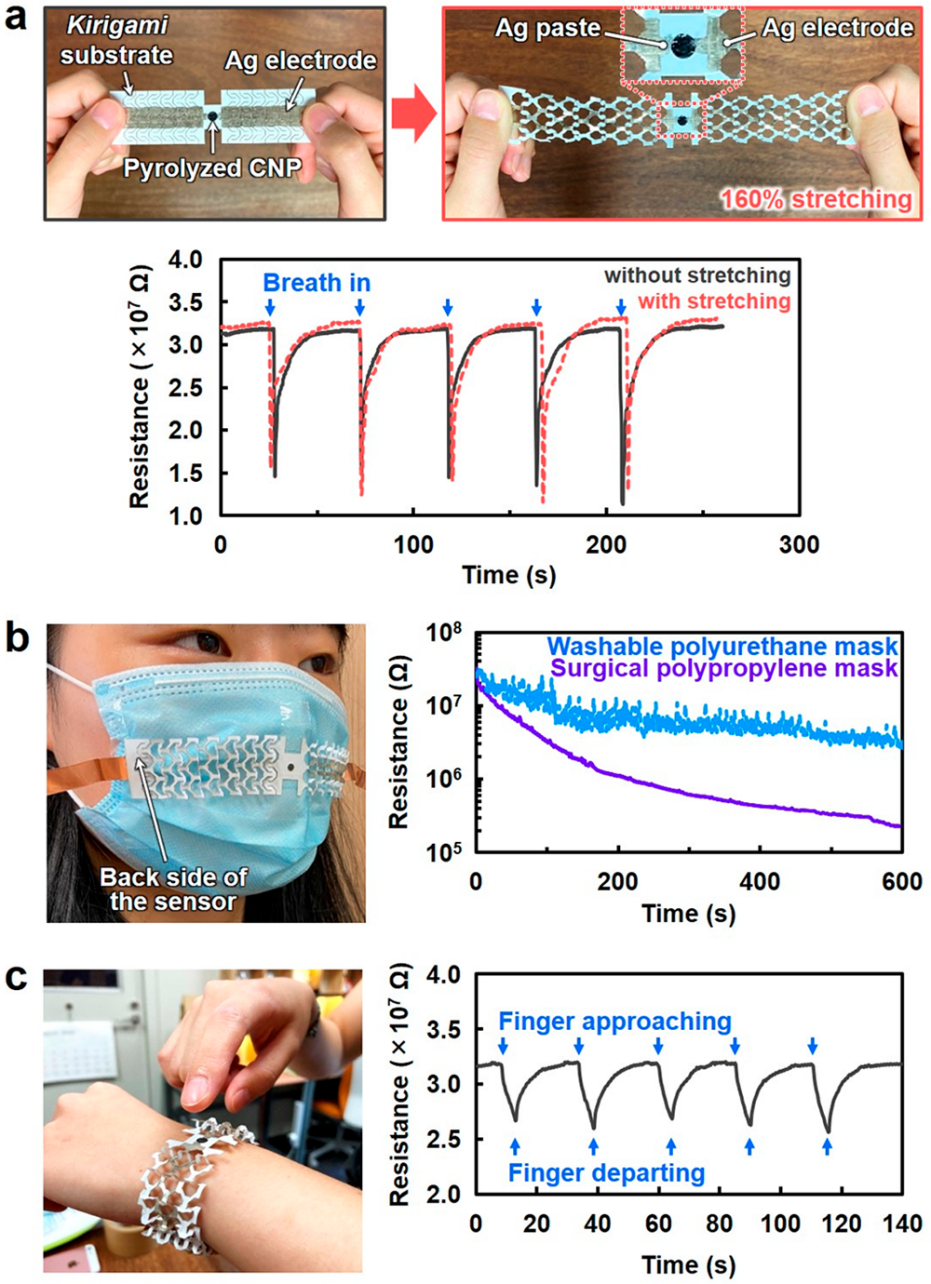
Stretchable and wearable sensor devices based on the pyrolyzed cellulose nanofiber paper (CNP) sensor and *kirigami* paper substrate. (a) Optical images of the sensor device and water vapor sensing in exhaled human breath before and after stretching, demonstrating the (b) monitoring of human exhalation-derived water vapor leaked from the face masks and (c) detection of skin moisture upon an approaching finger. The finger approaches close to ∼5 mm to the pyrolyzed CNP sensor and is held for 5 s, followed by departing away from the sensor.

Pyrolyzed CNP was further applied to wearable devices by designing macroscopic device configurations (Figure 5). A circular-shaped pyrolyzed CNP sensor was mounted on a stretchable *kirigami* paper substrate with patterned Ag electrodes (Figure 5a). The sensor response of the device to human-exhalation-derived water vapor remained almost unchanged, even with a stretching of 160%. The device also showed sufficient mechanical stability enough to maintain its sensing performance even after 100-cycle stretching (Figure S19). Then, the facial mask with the stretchable sensor device was worn, and the response was monitored through respiration (Figure 5b). Pulsating sensor responses corresponding to respiration were observed together with a slight decrease in the base resistance upon using a washable polyurethane mask, while only a gradual decrease in sensor resistance was observed upon using a surgical polypropylene mask. The results were consistent with the better water-vapor-capturing property of a surgical mask than that of a washable mask.^42^ Additionally, a stretchable sensor device was worn on a wrist (similar to a watch), and the skin moisture was monitored (Figure 5c). Clear sensor responses were reproduced with an approaching finger. These results suggested the potential use of wearable pyrolyzed CNP sensor for applications such as medical diagnosis of dry mouth,^43^ healthcare/skincare, and environmental monitoring.^44^ Thus, the applicability of the pyrolyzed CNP sensor to stretchable and wearable devices was successfully demonstrated, proving that its trans-scalable structural designability provides high functionality and various device configurations for versatile utilization.

### Application to Conductor Electrode for Enzymatic Glucose Biofuel Cell

**Figure 6:**
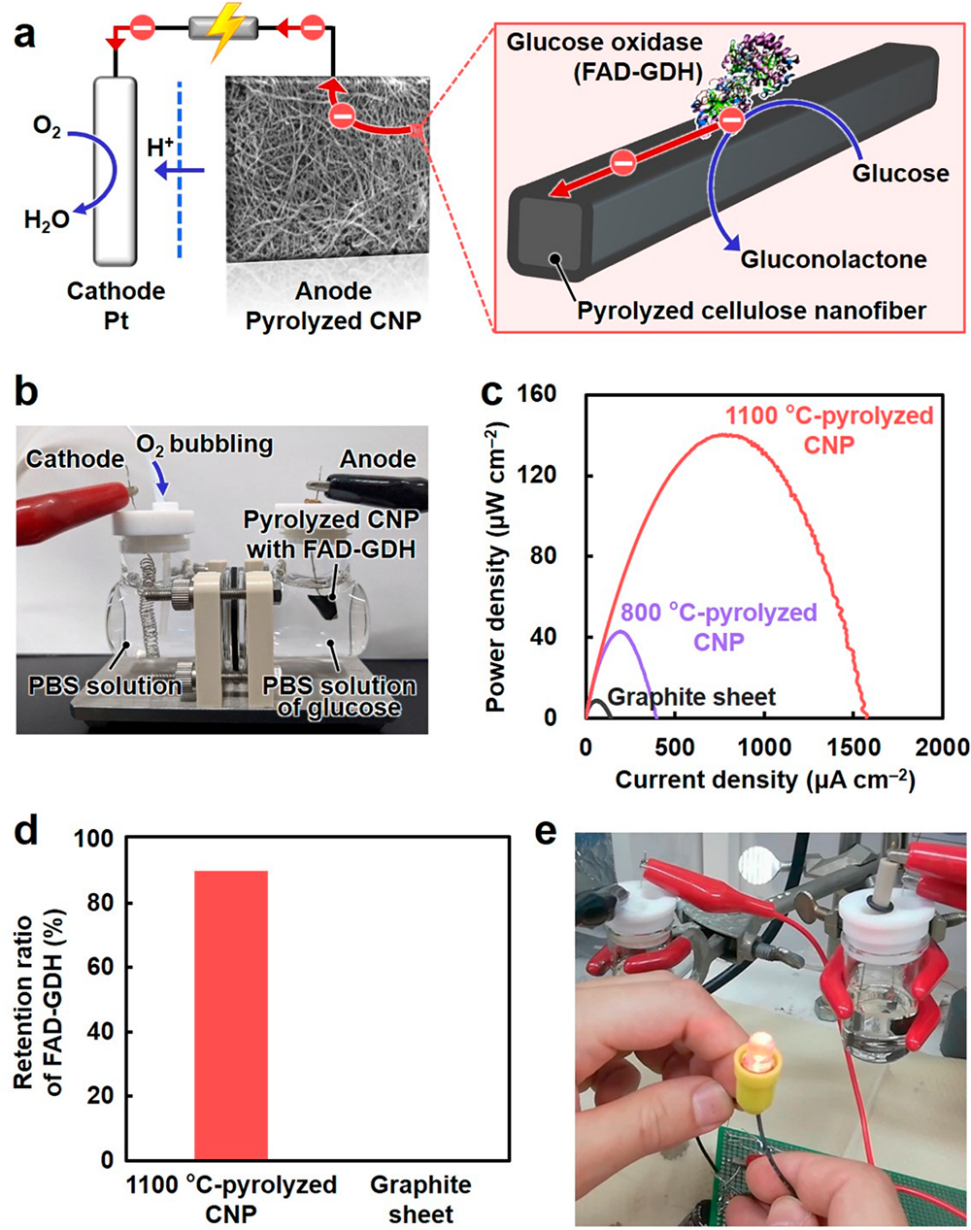
Biocatalytic electrode performance of pyrolyzed cellulose nanofiber paper (CNP) for an enzymatic glucose biofuel cell. (a) Schematic illustration and (b) optical image of an enzymatic glucose biofuel cell using flavin adenine dinucleotide-dependent glucose dehydrogenase (FAD-GDH) immobilized on the pyrolyzed CNP anode in a phosphate buffered saline (PBS) solution of d-glucose. (c) Power density as a function of current density for the CNPs pyrolyzed at 800 and 1100 °C and the graphite sheet. (d) Retention ratio of the FAD-GDH adsorbed on the CNP pyrolyzed at 1100 °C and graphite sheet after stirring in water for 2 h. (e) Lighting of a red LED using the energy generated by two membrane-less biofuel cells by employing the CNP pyrolyzed at 1100 °C as both the anode and cathode.

To demonstrate the significance of wide tunability of electrical properties, pyrolyzed CNP was used as an anode for an enzymatic glucose biofuel cell for energy generation (Figure 6a,b). In this application, the anode should immobilize the glucose oxidase enzyme and conduct electrons that are extracted from glucose by the immobilized enzymes.^45^ Accordingly, the CNPs pyrolyzed at 800 and 1100 °C, with nanoscale pores and quasi-conductor properties, were evaluated as anodes. Flavin adenine dinucleotide-dependent glucose dehydrogenase (FAD-GDH) was used as the glucose oxidase enzyme. The CNP pyrolyzed at 1100 °C afforded a 3.5-times higher power density (∼140 μW cm^–2^) than the CNP pyrolyzed at 800 °C (∼40 μW cm^–2^; Figure 6c). The CNP pyrolyzed at 1100 °C showed a higher electrical conductivity (lower resistivity: 4.5 × 10^–2^ Ω cm) than that pyrolyzed at 800 °C (1.9 × 10^–1^ Ω cm), while there was no significant difference in their surface areas (Figures S2 and S3). Therefore, the higher electrical conductivity of the CNP pyrolyzed at 1100 °C could enhance the power density, *i*.*e*., energy generation, by improving the conduction of electrons extracted from glucose. Tailoring the 3D network nanostructures of pyrolyzed CNP also contributed to an increase in its power density; pyrolyzed CNP with pore sizes of <100 nm (Figure 1d) afforded a high power density while that with a densely packed nanostructure showed poor power density (Figure S20). Furthermore, the CNP pyrolyzed at 1100 °C with porous nanostructures showed a significantly higher power density than the commercial graphite sheet with a flat surface structure and high electrical conductivity (resistivity of the order of 10^–5^ Ω cm). The CNP pyrolyzed at 1100 °C retained ∼90% of the adsorbed FAD-GDH after stirring in water for 2 h, while the commercial graphite sheet showed desorption of almost all the adsorbed FAD-GDH (Figure 6d and Figure S21). Therefore, pyrolyzed CNP with a porous nanostructure allowed for efficient immobilization of the FAD-GDH enzyme, affording a high power density. The CNP pyrolyzed at high temperature containing tailored 3D network porous nanostructures acted as a high-performance anode for an enzymatic glucose biofuel cell by affording high electrical conductivity and efficient enzyme-immobilization properties. The pyrolyzed CNP-based biofuel cells generated sufficient energy to light an LED (Figure 6e). Thus, the wide tunability of the electrical properties of pyrolyzed CNP semiconductors can expand their functionality and applicability for various electronic devices.

## Conclusion

In summary, 3D structural design and tuning of the electrical conduction of a pyrolyzed CNP semiconductor are described. Both the nano–micro–macro trans-scale designability of 3D structures (in the nanometer–centimeter range in terms of pore size, surface structure, and macroscopic morphology) and wide tunability of electrical resistivity (in the 10^12^–10^–2^ Ω cm range) are notable features of the pyrolyzed CNP semiconductor that are better than those of previously reported 3D semiconductor structures. Thus, the pyrolyzed CNP semiconductor can allow customization of its structure and functions according to the desired use, thereby affording broad applicability as a wearable water-vapor-selective sensor and an enzymatic biofuel cell electrode for energy generation. The electrical and chemical properties of the pyrolyzed CNP semiconductor can be potentially further modulated by modifying its molecular structures using various methods such as heteroatom doping. This study can be a milestone in manipulating the functionality and practicality of semiconducting nanomaterials for various electronic applications. As pyrolyzed CNP semiconductors can be prepared from ubiquitous and abundant biological resources, this strategy can contribute toward the realization of sustainable electronics.

## Methods

### Materials

Cellulose nanofibers with a width of 22 ± 8 nm and carboxylate content of 0.08 ± 0.02 mmol g^–1^ were prepared from never-dried pulp (softwood bleached kraft pulp) by employing the method described in our previous report.^46^ First, an aqueous suspension of the pulp (0.3 wt %, 2 L) was treated using a high-pressure water-jet system equipped with a counter-collision chamber (Star Burst, HJP-25005E, Sugino Machine Co., Ltd., Uozu, Japan). The pulp suspension was ejected from a nozzle with a diameter of 0.10 mm under a high pressure of 245 MPa with 100 passes. Iodine (>99.8% purity), *t*-BuOH (>99.0% purity), and disodium hydrogen phosphate (>99.0% purity) were obtained from Nacalai Tesque, Inc. (Kyoto, Japan). Acetonitrile, sodium dihydrogen phosphate (>99.0% purity), and bilirubin oxidase (from *Myrothecium* sp., BOD) were purchased from FUJIFILM Wako Pure Chemical Corporation (Osaka, Japan). d-Glucose (>98.0% purity) and 1,4-naphthoquinone (>98.0% purity) were purchased from Tokyo Chemical Industry Co., Ltd. (Tokyo, Japan). Nafion and graphite sheets (Graphinity) were obtained from The Chemours Company FC, LLC and Kaneka Corp. (Osaka, Japan), respectively. All other chemicals were of reagent grade and used without further purification.

### Spatially Controlled Drying of Cellulose Nanofiber Dispersions for the Preparation of CNP

An aqueous dispersion of cellulose nanofibers (0.2 wt %, 200 mL) was dewatered by suction filtration through a membrane filter (H020A090C, hydrophilic polytetrafluoroethylene (PTFE) membrane, pore diameter of 0.1 μm, Advantec Toyo Kaisha, Ltd., Tokyo, Japan). *t*-BuOH (200 mL) was then poured into it and gently filtered, followed by the peeling of the wet sheet from the filter. The wet sheet was freeze-dried overnight (FDU-2200, Tokyo Rikakikai, Co., Ltd., Tokyo, Japan) to prepare CNP with porous nanostructures. CNP with densely packed nanostructures was prepared without *t*-BuOH treatment by hot-press drying at 110 °C for 15 min (1.1 MPa) instead of freeze-drying. CNP with honeycomb-like porous microstructures was prepared using an aqueous dispersion of cellulose nanofibers (∼1.0 wt %) *via* the unidirectional freeze-drying technique.^30^

### Embossing, *Origami*, and *Kirigami* Processing of CNP

An aqueous dispersion of cellulose nanofibers (0.2 wt %, 200 mL) was dewatered by suction filtration through a membrane filter. *t*-BuOH (200 mL) was then poured into it and gently filtered. For embossing, the obtained wet sheet was pressed with an imprint mold (DTM-1-3, KYODO INTERNATIONAL, Inc., Kanagawa, Japan) at 25 °C for 10 min (10 MPa), followed by hot-press drying at 110 °C for 15 min (10 MPa) and peeling from the filter. For *origami* processing, the CNP prepared by *t*-BuOH treatment followed by hot-press drying at 110 °C for 15 min (1.1 MPa) without the imprint mold was used. For *kirigami* processing, the CNP was cut into different geometries using a hole puncher or a CO_2_ laser cutting machine (HAJIME CL1 PLUS, Oh-Laser Co., Ltd., Saitama, Japan) before use.

### Pyrolysis of CNP

Prior to pyrolysis, CNP was pretreated with iodine vapor (same weight as of CNP) in a sealed flask at 100 °C for 24 h. Thereafter, the resulting paper was pyrolyzed in a furnace under nitrogen gas supplied at a flow rate of 500 mL min^–1^ (KDF-75, DENKEN-HIGHDENTAL Co., Ltd., Kyoto, Japan) to suppress rapid pyrolysis *via* autoxidation^47^ and remove the generated corrosive HI gas in the furnace. The paper samples were thermally treated in three stages: (1) increase in temperature from room temperature to 240 °C at a rate of 2 °C min^–1^ and holding for 17 h; (2) increase in the temperature to peak temperatures ranging from 300–1100 °C at a rate of 2 °C min^–1^ and holding for 1 h; (3) cooling to room temperature at a rate of 2 °C min^–1^.

### Evaluation of Electrical Properties of Pyrolyzed CNP

The electrical properties of pyrolyzed CNP were evaluated *via* four-probe current–voltage (*I*–*V*) measurements using a semiconductor parameter analyzer (4200SCS, Keithley Instruments, Inc.) with a probe station. Prior to the measurements, Pt electrodes (width *W* = 3 mm, gap *L* = 500 μm, thickness *t* = 500 nm) for contacting the probes were deposited on pyrolyzed CNP by radio frequency sputtering with a metal mask. The electrical measurements were performed by varying the ambient temperature *T* from room temperature to 200 °C under a vacuum of <1.0 × 10^–2^ Pa. The electrical resistivity ρ of pyrolyzed CNP was estimated using the equation: ρ = *R*_four_*Wd*/*L*, where *R*_four_ is the four-probe resistance and *d* is the thickness of pyrolyzed CNP. The activation energy *E*_a_ of electrical conductivity was estimated from the *ρ–T* characteristics using the Arrhenius equation: 1/ρ = *A* exp(−*E*_a_/*k*_B_*T*), where *A* is a constant and *k*_B_ is the Boltzmann constant. The electrical resistivity values of CNP and pyrolyzed CNP at 300, 350, and 400 °C were also measured using a resistivity meter with a ring-type probe (Hiresta-UX, MCP-HT800, Mitsubishi Chemical Analytech Co., Ltd., Chigasaki, Japan). The optical bandgap values were calculated from the ultraviolet–visible–near-infrared (UV–vis–NIR) absorption spectra using Tauc’s equation:^48^ (*αhν*)^1/*n*^ = *A*(*hν* −*E*_g_), where α, *hν*, *A*, and *E*_g_ are the absorbance, photon energy, constant, and optical bandgap, respectively. The parameter *n* is 1/2 and 2 for the direct and indirect transitions, respectively. The optical bandgap was determined by plotting (*αhν*)^1/*n*^ versus photon energy (*hν*) and extrapolating the linear region of the curve to the *X*-axis. The optical bandgap values of the original crystalline cellulose nanofiber and amorphous pyrolyzed cellulose nanofiber were estimated to be *n* = 1/2 and 2, respectively (Figures S7–S9). The Hall effect measurements with correction for the thermomagnetic effects^49^ and resistivity measurements by the Van der Pauw method^50^ were performed to evaluate the carrier concentration, type, and mobility of pyrolyzed CNP using a customized Hall effect measurement system (Nagase Techno-Engineering Co., Ltd., Tokyo, Japan) with a semiconductor parameter analyzer (4200SCS, Keithley Instruments, Inc.) or a ResiTest8330 system (TOYO Corp., Tokyo, Japan) in DC mode at room temperature.

### Evaluation as Water Vapor Sensors

The water vapor sensing performance of the CNP with 3D porous nanostructures pyrolyzed at 600 °C was evaluated using a previously reported method.^41^ Briefly, the sensor device using pyrolyzed CNP was assembled on an alumina (Al_2_O_3_) substrate (width, 13 mm; length, 9 mm; thickness, 0.5 mm) after comb-type Au electrodes (width *W* = 200 μm, length *L* = 3.4 mm, gap *D* = 200 μm) were sputtered on the paper. Au electrodes were connected to Pt wires using Ag paste. The sensor device was then placed in the measurement apparatus. The measurement apparatus was equipped with a gas mixing system, electric furnace, and sensor measurement chamber to evaluate the electrical resistance in various atmospheres. Water vapor was introduced into the atmosphere by blowing in deionized water using synthetic air, and the exact humidity was determined using a commercial capacitance-type humidity sensor (TR-77Ui, T&D Corp., Matsumoto, Japan). In addition, to evaluate gas selectivity, the sensing performances for gases such as 1000 ppm of CO_2_ or 20 ppm EtOH in synthetic air, pure oxygen, and pure nitrogen were determined. The flow rate of the sample gases was adjusted to 100 cm^3^ min^–1^ using a mass flow controller (SEC-series, HORIBA STEC, Co., Ltd., Kyoto, Japan). The sensor device was connected to the standard resistance, and the voltage across the standard resistance was measured under an applied DC of 1 V to estimate the electrical resistance of the sensor device. The applied voltage was determined within the range where the temperature of the device does not change by Joule heating (Figure S22). The voltage across the standard resistance was measured using an electrometer (2701, Keithley Instruments, Inc.). The stretchable paper device was fabricated using pyrolyzed CNP, Ag paste (DOTITE, FA-451A, FUJIKURA KASEI Co., Ltd., Tokyo, Japan), Ag circuit marker, and circuit paper (TK-632877-01 and TK-632893-01, TAKEO Co., Ltd., Tokyo, Japan), and its water vapor sensing performance was evaluated using an electrochemical workstation (ModuLab XM ECS equipped with a Femto Ammeter card, Solartron Analytical-AMETEK Advanced Measurement Technology Inc., Wokingham Berkshire, UK). Informed consent was obtained from all participants for the experiment of human-exhalation-derived water vapor sensing and wearable demonstrations.

### Evaluation as an Electrode for Enzymatic Glucose Biofuel Cells

For the biofuel cell, the anode was fabricated by connecting the CNP pyrolyzed at 800 or 1100 °C (1 cm × 1 cm) and a platinum wire with a conductive adhesive. A graphite sheet was used as an anode for comparison. One side of the anode was covered with an insulating epoxy resin. 1,4-Naphthoquinone as a mediator dissolved in acetonitrile (0.1 mmol mL^–1^, 100 μL) was dropped onto the electrode using a pipet gun and dried in air. Subsequently, 10 mg of FAD-GDH was applied to the electrode surface and dried at room temperature *in vacuo* for 1 h. A curled platinum wire was used as the cathode. The anode and cathode were separated using the Nafion membrane, and phosphate buffer (PBS, pH = 7, 25 mL) was added to the cathode side, while a PBS solution of d-glucose (1 mol L^–1^, 25 mL) was added to the anode side. O_2_ bubbling was also employed on the cathode side. The power curves were obtained by scanning the voltage between the open-circuit voltage of the cell to 0 V at a constant scan rate of 1 mV s^–1^ using an electrochemical measurement system (SI1287, Solartron Analytical, Hampshire, UK).

### Evaluation of FAD-GDH-Immobilization Performance

Pyrolyzed CNP or graphite sheet (5 mg) was immersed in an aqueous solution (Milli-Q water) of FAD-GDH (3.3 mg mL^–1^, 1 mL), followed by stirring for 1 h at room temperature and letting it stand at 4 °C for 19 h. Thereafter, filtration through a hydrophilic PTFE membrane filter (Millex-LG, pore size: 0.2 μm, Merck Millipore Ltd., Darmstadt, Germany) was performed, and the filtrate was subjected to UV–vis analysis (V-670ST, JASCO Corp., Tokyo, Japan). The adsorption ratio of FAD-GDH onto the pyrolyzed CNP or graphite sheet was estimated by comparing the absorbance of the filtrate at 276 nm to that of the original FAD-GDH solution. Pyrolyzed CNP or graphite sheet with adsorbed FAD-GDH was recovered by filtration through a hydrophilic PTFE membrane filter (Omnipore, pore size: 1.0 μm, Merck Millipore Ltd., Darmstadt, Germany), followed by immersion in 1 mL of Milli-Q water. After stirring for 2 h at room temperature, filtration through a hydrophilic PTFE membrane filter (Millex-LG, pore size: 0.2 μm) was performed, and the filtrate was subjected to UV–vis analysis to confirm FAD-GDH release. The FAD-GDH retention ratio was estimated by considering the released FAD-GDH from the one-time-adsorbed counterpart on pyrolyzed CNP or graphite sheet.

### LED Lighting Test Using the Pyrolyzed CNP-Based Biofuel Cells

Both the anode and cathode were fabricated using the CNP pyrolyzed at 1100 °C by employing the same method. 1,4-Naphthoquinone (0.1 mmol mL^–1^, 100 μL) and FAD-GDH (10 mg) were cast onto the anode, and BOD (2 mg) was cast onto the cathode. The membrane-less biofuel cell was assembled by immersing the anode and cathode in a PBS solution containing glucose. For the red LED (1.5 V, EK Japan Co. Ltd., Fukuoka, Japan) lighting test, a commercial capacitor (16 V, 6800 μF, Nippon Chemi-con Corp., Tokyo, Japan) and two biofuel cells were connected in series.

### Analyses

UV–vis–NIR absorption spectra were recorded at room temperature using a V-770 spectrophotometer (JASCO Corp., Tokyo, Japan). The paper samples were thoroughly crushed and added to distilled water, sonicated for 2 h, and then centrifuged at 12 000 rpm for 1 min. The resulting supernatant was used for UV–vis–NIR absorption analysis. The surface was analyzed using an FE-SEM system (SU-8020, Hitachi High-Tech Corp., Tokyo, Japan). HR-TEM observations were performed using a JEM-ARM 200F instrument (JEOL Ltd., Tokyo, Japan). The specific surface areas were determined by nitrogen adsorption measurements using a NOVA 4200e BET instrument (Quantachrome Instruments, Kanagawa, Japan). Elemental analyses were conducted using a 2400II instrument (PerkinElmer Japan Co. Ltd., Kanagawa, Japan). Iodine content was measured by the modified Stragand–Safford method^51^ using a halogen and sulfur analyzer (YANAGIMOTO MFG. Co. Ltd., Kyoto, Japan). XRD patterns were recorded using an Ultima IV system (Rigaku Corp., Tokyo, Japan) with Ni-filtered Cu Kα radiation (1.5418 Å) and a scanning angle (2θ) range of 5–80° at 30 kV and 40 mA. The crystallite sizes of the graphene fragments in the in-plane (*L*_a_) and stacking (*L*_c_) directions were calculated from the XRD spectra using a Scherrer-type formula: *L* = *kλ*/β cos θ, where λ, β, and θ are the X-ray wavelength, full width at half-maximum, and Bragg angle, respectively. *L*_a_ and *L*_c_ were estimated from the (10) and (002) reflections with *k* = 1.84 and 0.89, respectively, according to the method described in previous reports.^52^ FT-IR/attenuated total reflection spectra were obtained using a KJP-05120S instrument (PerkinElmer Japan Co. Ltd., Kanagawa, Japan). Laser Raman spectroscopic analyses were conducted using a RAMAM-touch instrument (Nanophoton Corp., Osaka, Japan). Solid-state ^13^C NMR spectra were recorded on an Avance III 600WB instrument (Bruker Japan K. K., Tokyo, Japan). The samples were crushed and packed into a 4 mm rotor and then spun at 12 kHz.

## Supporting Information Available

Figures of characterization of the cellulose nanofibers used in this study, relative apparent volume and weight, and bulk density of the CNP pyrolyzed at different temperatures with I_2_ pretreatment, specific surface areas of the CNPs pyrolyzed at different temperatures with I_2_ pretreatment, FE-SEM images of the original CNP and that pyrolyzed at different temperatures, current versus voltage characteristics of the CNP pyrolyzed at 600 °C measured *via* two-probe method with Pt, Au, and Ag electrodes, optical bandgap values of the cellulose nanofibers pyrolyzed at different temperatures, Tauc plots and estimated optical bandgap values of the original and pyrolyzed cellulose nanofibers, Hall effect measurement for the CNP pyrolyzed at 650 °C, HR-TEM images of the cellulose nanofiber pyrolyzed at 300 and 1000 °C, molecular structures of pyrolyzed cellulose nanofiber, elemental contents of the cellulose nanofiber pyrolyzed at different temperatures, FT-IR spectra of the cellulose nanofiber pyrolyzed at different temperatures, putative mechanism for the drastic and progressive modulation of the electrical properties of cellulose nanofibers upon pyrolysis, water-vapor-selective sensing performance of the CNP pyrolyzed at 600 °C, water vapor sensing in human-exhaled breath using the CNP pyrolyzed at 600 °C, water vapor sensing in human-exhaled breath by a sensor device that integrated the pyrolyzed CNP sensor and *kirigami* paper substrate before and after 100-cycle of 160% stretching, biocatalytic electrode performance of the CNP pyrolyzed at 800 °C for an enzymatic glucose biofuel cell, FAD-GDH immobilization performances of the CNP pyrolyzed at 1100 °C and graphite sheet, and measurements of current density and temperature for the CNP pyrolyzed at 650 °C during applying voltage, table of comparison of pyrolyzed CNP to other materials with tunable electronic properties, and discussion of molecular structures of pyrolyzed CNP (PDF)
