# Supplementary material for: Nanocellulose Paper Semiconductor with a 3D Network Structure and Its Nano–Micro–Macro Trans-Scale Design
Source: ACS Nano. 2022 Apr 26;16(6):8630–40. doi: 10.1021/acsnano.1c10728 (PMC9245344; doi:10.1021/acsnano.1c10728)
Supplement: Supplementary file 1 — nn1c10728_si_001.pdf [file nn1c10728_si_001.pdf]

Supporting Information

Nanocellulose Paper Semiconductor with a 3D Network Structure and Its Nano-Micro-Macro Trans-Scale Design

Hirotaka Koga,[1,] Kazuki Nagashima,[2,3,]* Koichi Suematsu,[4] Tsunaki Takahashi,[2,3] Luting Zhu,[1] Daiki Fukushima,[1] Yintong Huang,[1] Ryo Nakagawa,[5] Jiangyang Liu,[2] Kojiro Uetani,[1] Masaya Nogi,[1] Takeshi Yanagida,[2,6] Yuta Nishina[7]*

[1]SANKEN (The Institute of Scientific and Industrial Research), Osaka University, 8-1 Mihogaoka, Ibaraki, Osaka 567-0047, Japan

[2]Department of Applied Chemistry, Graduate School of Engineering, The University of Tokyo, 7-3-1 Hongo, Bunkyo-ku, Tokyo 113-8656, Japan

[3]Japan Science and Technology Agency (JST), PRESTO, 4-1-8 Honcho, Kawaguchi, Saitama 332-0012, Japan

[4]Department of Advanced Materials Science and Engineering, Faculty of Engineering Sciences, Kyushu University, 6-1 Kasuga-Koen, Kasuga, Fukuoka 816-8580, Japan

[5]Graduate School of Natural Science and Technology, Okayama University, 3-1-1 Tsushimanaka, Kita-ku, Okayama 700-8530, Japan

[6]Institute for Materials Chemistry and Engineering, Kyushu University, 6-1 Kasuga-Koen,

Kasuga, Fukuoka 816-8580, Japan

[7]Research Core for Interdisciplinary Sciences, Okayama University, 3-1-1 Tsushimanaka, Kita-ku, Okayama 700-8530, Japan

*Corresponding Authors

Hirotaka Koga, E-mail: hkoga@eco.sanken.osaka-u.ac.jp

Kazuki Nagashima, E-mail: kazu-n@g.ecc.u-tokyo.ac.jp

Supporting Figures and Tables

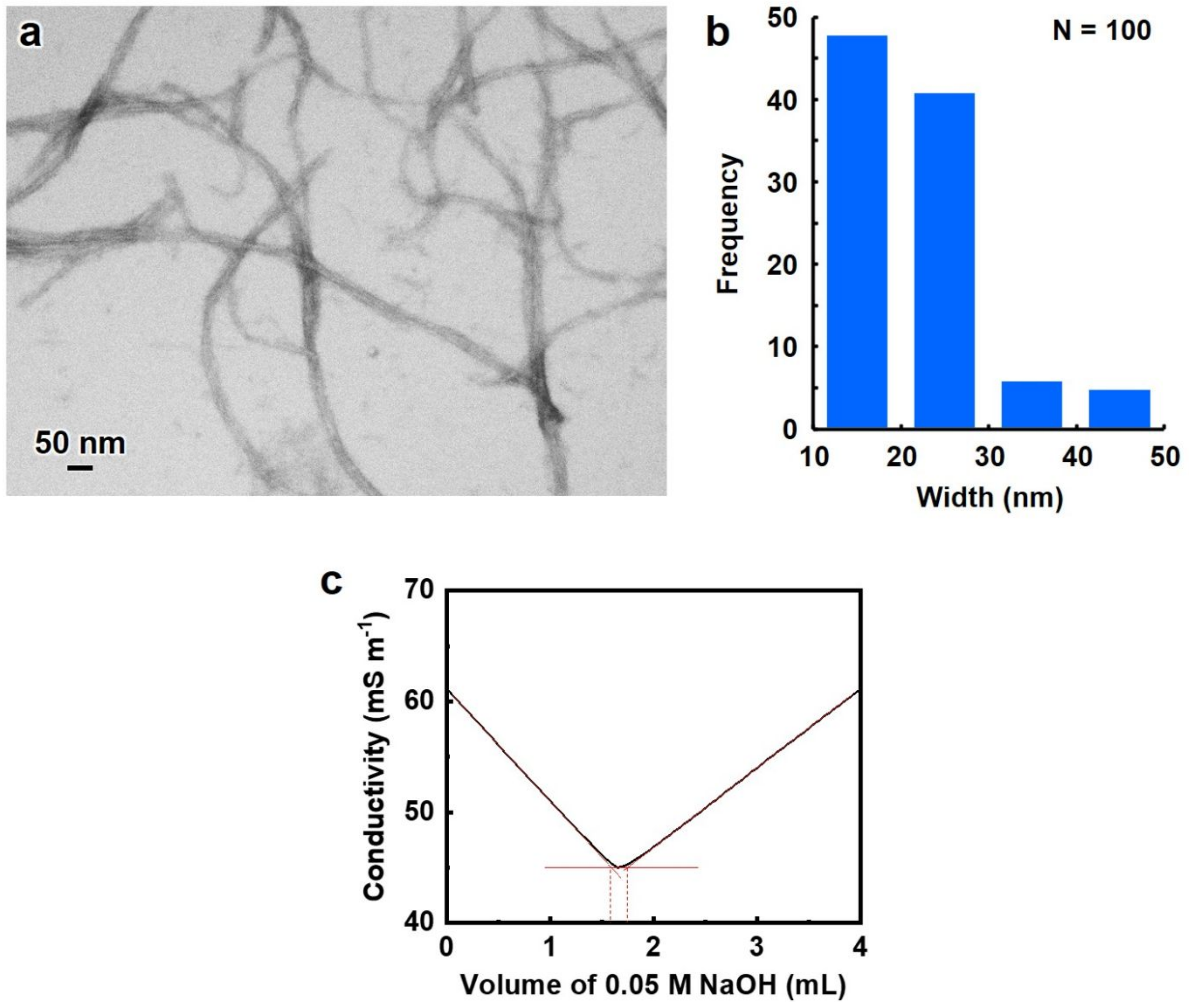

Figure S1. Characterization of the cellulose nanofibers used in this study. (a) A transmission electron microscopy (TEM) image and (b) a width distribution histogram of cellulose nanofibers, (c) conductometric curve of the titration of cellulose nanofibers using NaOH to calculate their carboxylate content.

Cellulose nanofibers were prepared from never-dried pulp (softwood bleached kraft pulp) by the aqueous counter collision method.[1] The as-prepared cellulose nanofibers were negatively stained with the phosphotungstic acid, and then subjected to TEM observation (**Figure S1a**). From the width distribution histogram of 100 individual cellulose nanofibers (**Figure S1b**), their average width was estimated as 22 ± 8 nm.

The carboxylate content of the cellulose fibers was measured by a conductometric titration method.[2] Briefly, a cellulose fiber suspension was diluted to 0.21 wt% with distilled water, followed by the addition of 0.01 M NaCl solution (5 mL). Subsequently, the pH of the suspension was adjusted to 2.5–3.0 by adding 0.5 M HCl. The suspension was then titrated by adding 0.05 M NaOH at a speed of 0.1 mL min^{-1} using an automatic titrator (AUT-701, DKK-TOA Corp., Tokyo, Japan). The carboxylate content (n, mmol g^{-1}) was calculated using the following equation:

$$n = \frac{V \times c}{\omega}$$

where V, c, and ω denote the volume of NaOH solution consumed in the plateau region of the titration profile (**Figure S1c**), concentration of the titrated NaOH solution, and weight of the measured sample, respectively. In this study, the calculated carboxylate content was 0.08 ± 0.02 mmol g^{-1}.

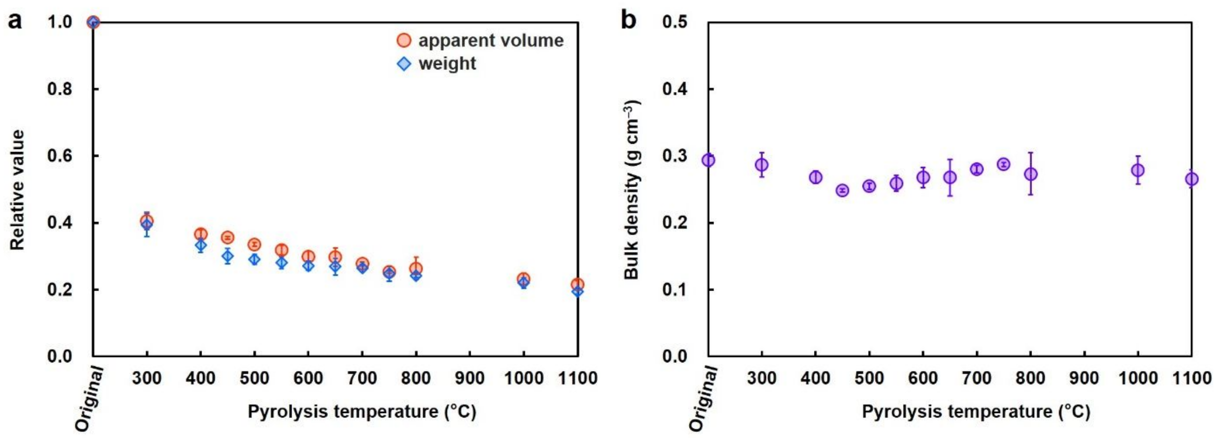

Figure S2. (a) Relative apparent volume and weight, and (b) bulk density of the cellulose nanofiber paper (CNP) pyrolyzed at different temperatures with I_2 pretreatment.

The apparent volume and weight of CNP with porous nanostructures decreased to ~22% and ~19%, respectively, after pyrolysis at 1100 °C (**Figure S2a**). The bulk density of the CNP was not largely changed by pyrolysis; the bulk density of the original CNP with porous nanostructures was changed from 0.294 to 0.266 g cm^{-3} after pyrolysis at 1100 °C (**Figure S2b**). Although CNP gradually shrank with an increase in the pyrolysis temperature (**Figure 1b**), it was freestanding even after pyrolysis at 1100 °C, allowing easy handling for evaluation and applications.

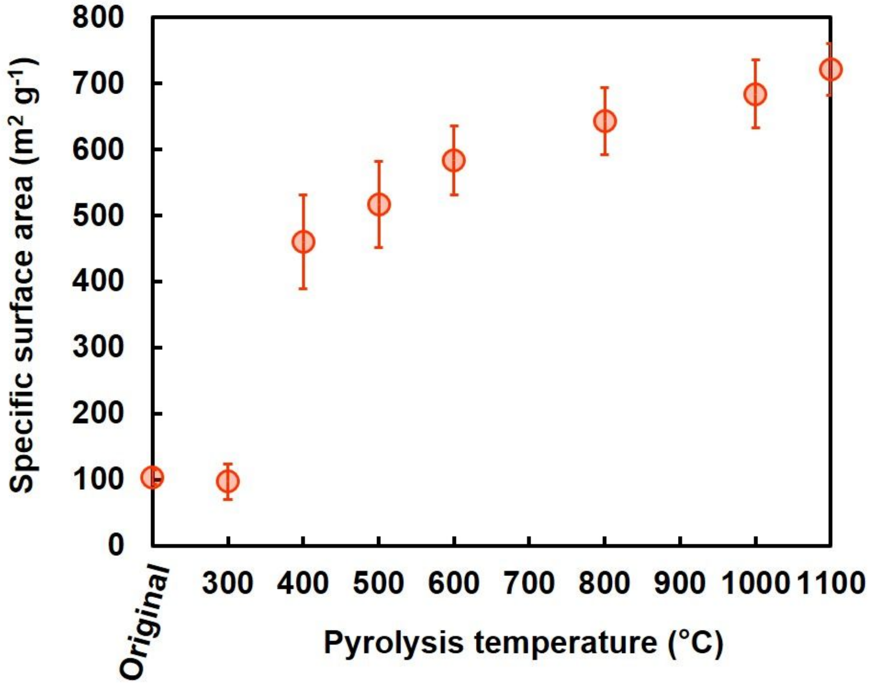

Figure S3. Specific surface areas of the cellulose nanofiber papers (CNPs) pyrolyzed at different temperatures with I_2 pretreatment.

CNP with porous nanostructures was prepared by solvent exchange with *tert*-butyl alcohol and freeze-drying and then pyrolyzed at different temperatures with I_2 pretreatment. The original morphology of cellulose nanofibers was maintained even after pyrolysis at 1100 °C (**Figure 1d**), while the specific surface area of pyrolyzed CNP increased (up to ~721 m^2 g^{-1}) with an increase in the pyrolysis temperature.

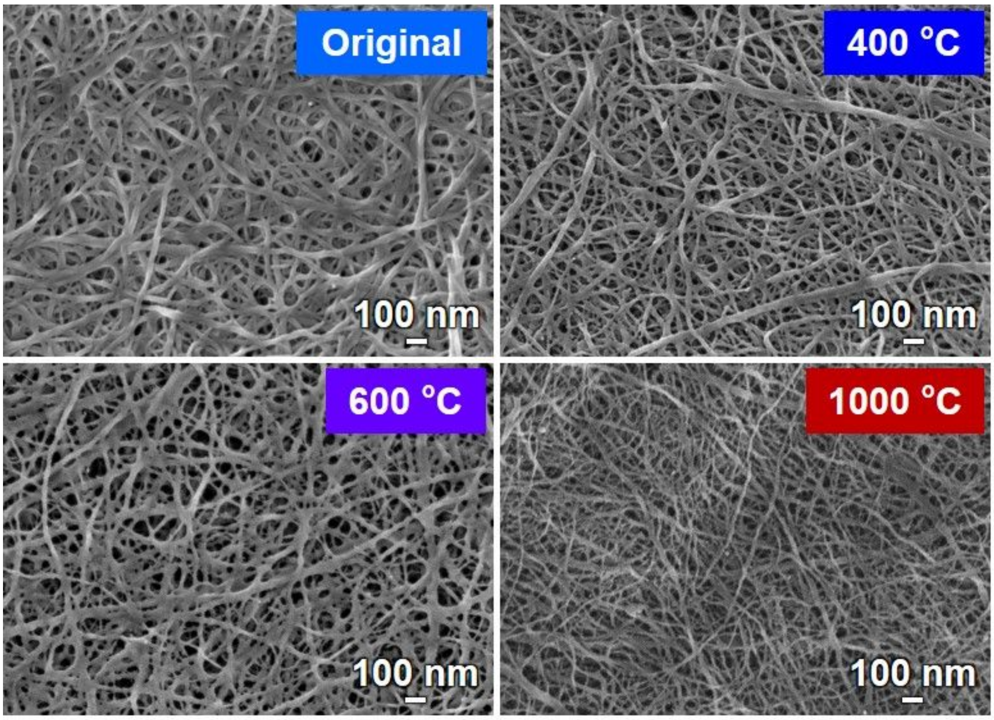

Figure S4. Field-emission scanning electron microscopy (FE-SEM) images of the original cellulose nanofiber paper (CNP) and that pyrolyzed at different temperatures.

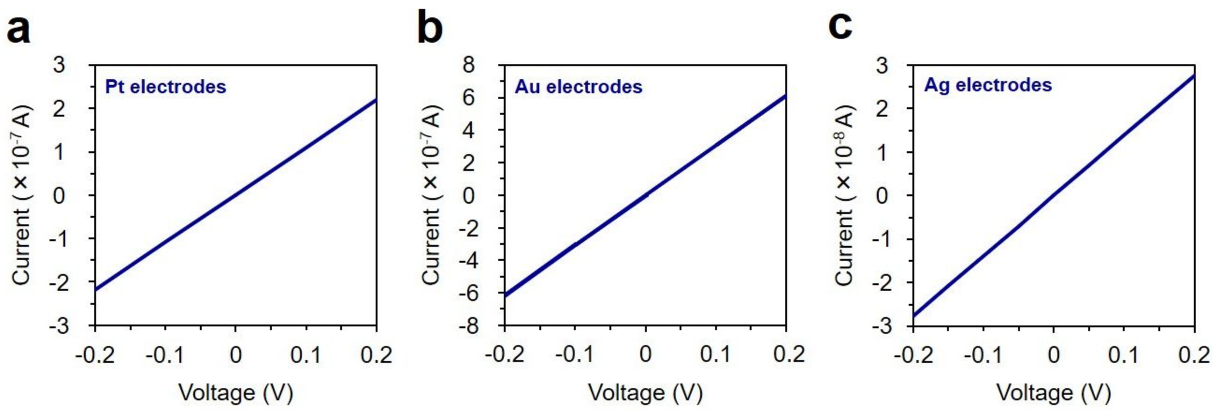

Figure S5. Current versus voltage characteristics of the cellulose nanofiber paper (CNP) pyrolyzed at 600 °C measured *via* two-probe method with (a) Pt, (b) Au, and (c) Ag electrodes. Measurement temperature: 293 (Pt and Ag) or 294 K (Au).

The pyrolyzed CNP showed a linear relationship in current−voltage characteristics for Pt, Au, and Ag electrodes, indicating that ohmic contact was formed between the pyrolyzed CNP and the electrodes.

Table S1. Comparison of pyrolyzed CNP to other materials with tunable electronic properties.

	Electrical resistivity (Ω cm)	Ref.
Pyrolyzed 3D CNP	10^{12}–10^{-2}	This work
Chemical-vapor-deposited 3D graphene foam	10^{-1}–10^{-2}	3
Density-controlled 3D graphene monolith	10^{2}–10^{0}	4,5
Density-controlled 3D carbon nanotube foam	10^{1}–10^{-1}	6
Doped 3D metal–organic framework thin film	10^{7}–10^{1}	7
Doped 3D covalent organic framework pellet	10^{7}–10^{2}	8
Doped 3D porous organic polymer pellet	10^{8}–10^{4}	9
Doped ZnSe nanowire	10^{9}–10^{-1}	10
Doped Si	10^{3}–10^{-4}	11
Doped GaAs	10^{0}–10^{-4}	11
Doped graphitic carbon nitride	10^{11}–10^{7}	12,13
Oxidized graphite	10^{3}–10^{-3}	14
Reduced graphene oxide	10^{3}–10^{-3}	14,15

CNP, cellulose nanofiber paper.

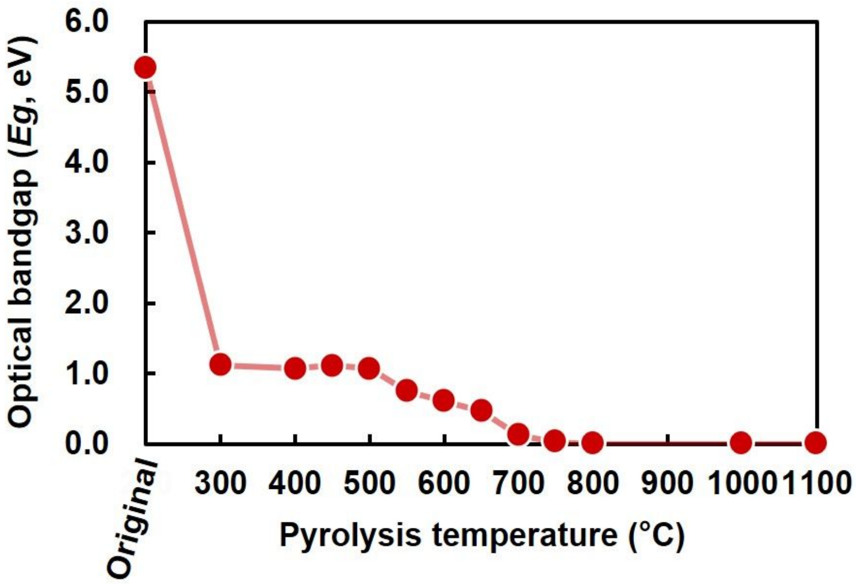

Figure S6. Optical bandgap values of the cellulose nanofibers pyrolyzed at different temperatures.

The bandgap values of the original and pyrolyzed cellulose nanofibers were optically estimated from their ultraviolet–visible-–near-infrared (UV–vis–NIR) absorption spectra using the Tauc plots[16] (see **Figure S7–9** for more details).

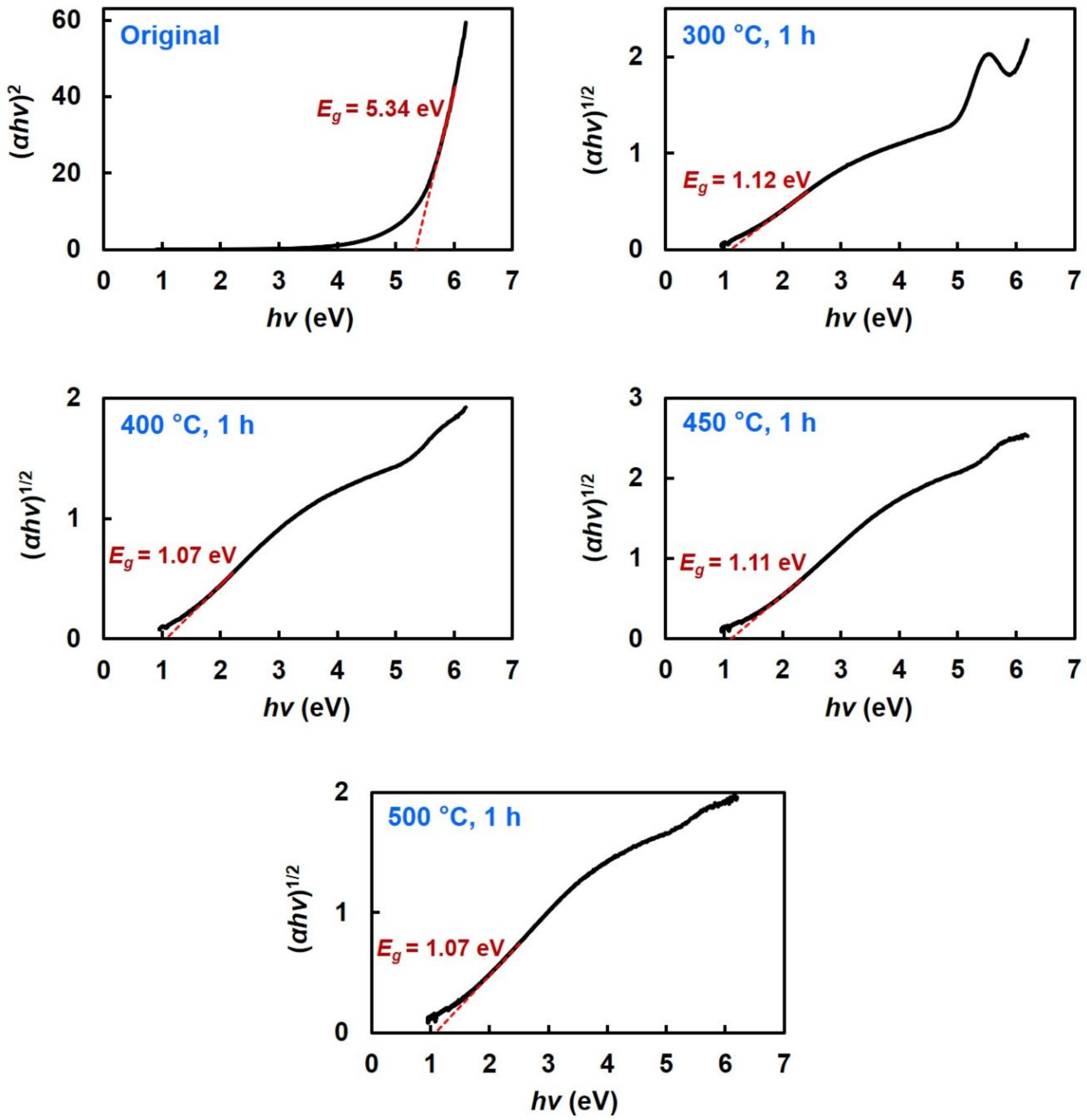

Figure S7. Tauc plots and estimated optical bandgap (E_g) values of the original and pyrolyzed cellulose nanofibers. Pyrolysis temperature: 300–500 °C.

The high optical bandgap of the original cellulose nanofiber (5.34 eV) decreased to ~1.12–1.07 eV after pyrolysis at 300–500 °C.

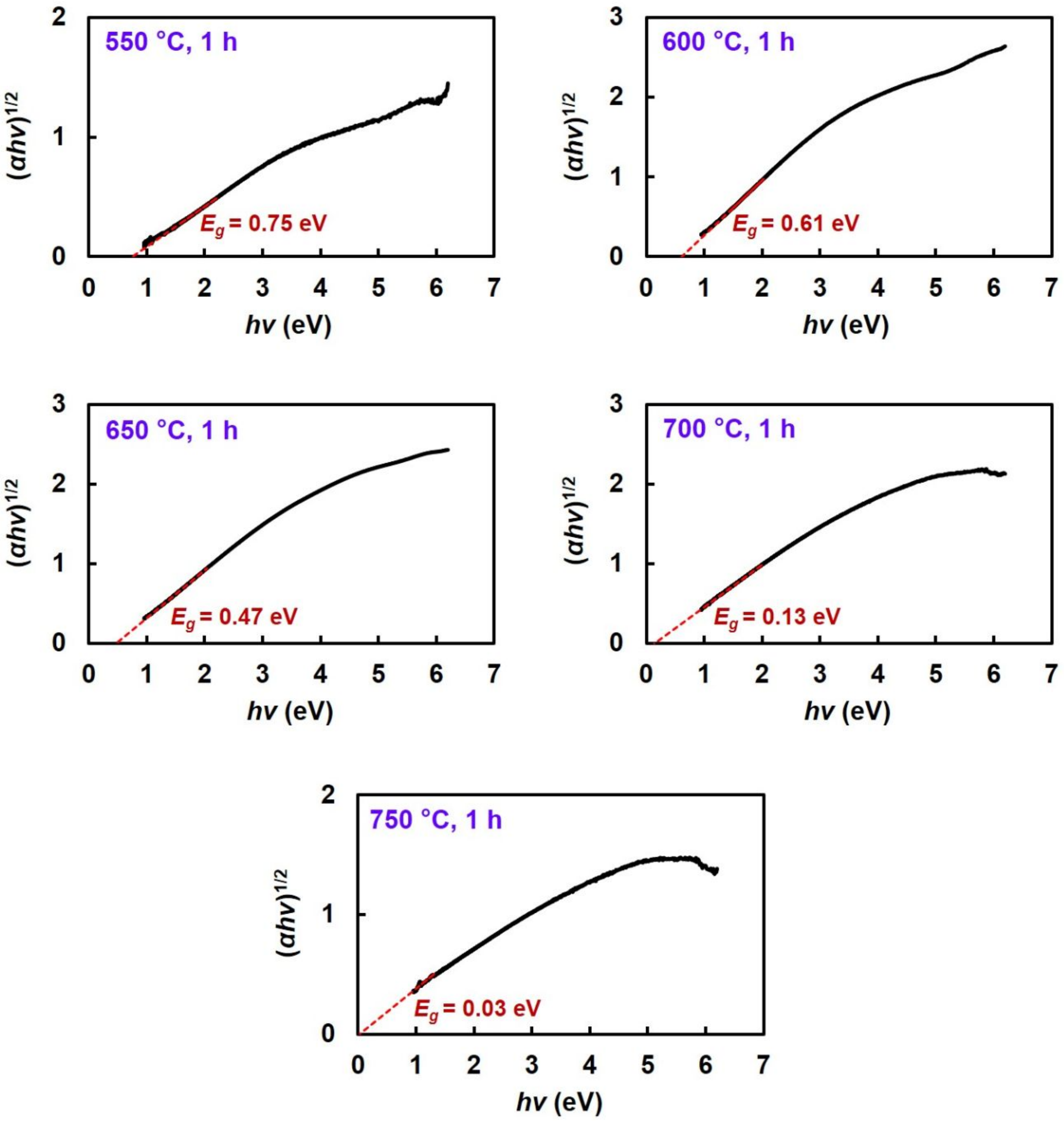

Figure S8. Tauc plots and estimated optical bandgap values of pyrolyzed cellulose nanofibers. Pyrolysis temperature: 550–750 °C.

Upon an increase in the pyrolysis temperature from 550 to 750 °C, the optical bandgap decreased from 0.75 to 0.03 eV.

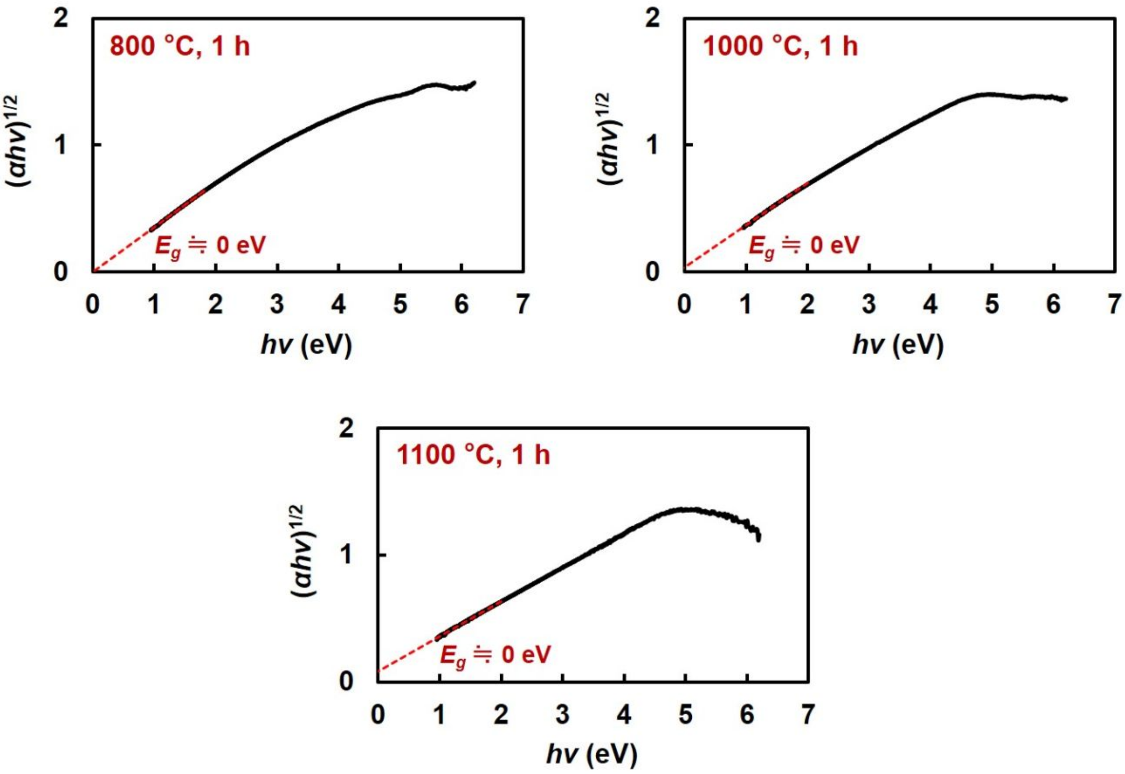

Figure S9. Tauc plots and estimated optical bandgap values of pyrolyzed cellulose nanofibers. Pyrolysis temperature: 800–1100 °C.

Tauc plots of the cellulose nanofiber pyrolyzed at 800–1100 °C had no cross point with the X-axis, suggesting a very small bandgap (almost 0 eV).[17] The CNPs pyrolyzed at temperatures of >800 °C with very low bandgaps exhibited conductor-like electrical conduction.

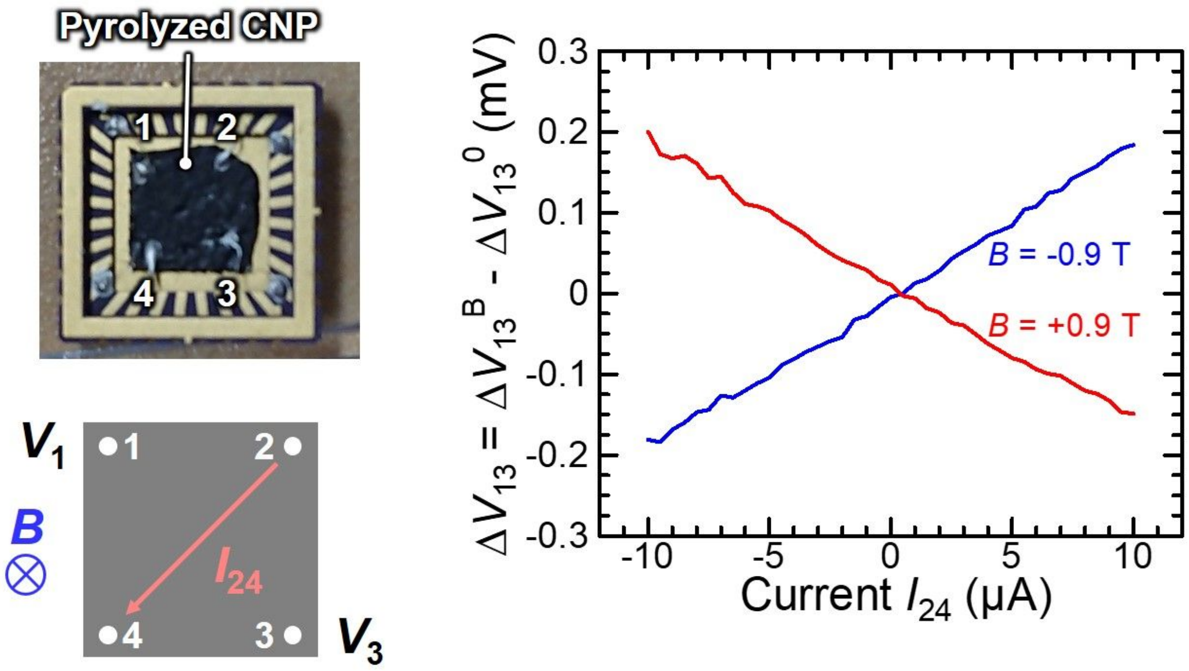

Figure S10. Hall effect measurement for the cellulose nanofiber paper (CNP) pyrolyzed at 650 °C. Paper thickness: 109 µm.

The Hall effect measurements with the correction for thermomagnetic effects[18] and resistivity measurements by the Van der Pauw method[19] for the pyrolyzed CNPs were performed to evaluate their carrier concentration, type, and mobility. The CNPs pyrolyzed at <650 °C could not be evaluated possibly due to their relatively high electrical resistivity. In contrast, the CNP pyrolyzed at 650 °C was successfully evaluated as follows. Negative ΔV_{13} for the positive magnetic flux density (B) and positive current (I_{24}) indicated electron transport, suggesting that the CNP pyrolyzed at 650 °C was an *n*-rich semiconductor. By assuming that pyrolyzed CNP had a uniform film structure, it was roughly estimated that the electrical resistivity, carrier mobility, and carrier concentration of the CNP pyrolyzed at 650 °C were 833 Ω cm, 2.59 cm^2 V^{-1} s^{-1}, and 2.89 × 10^{15} cm^{-3}, respectively. The CNPs pyrolyzed at 750, 1000, and 1100 °C were also evaluated in a similar manner (**Table 1**).

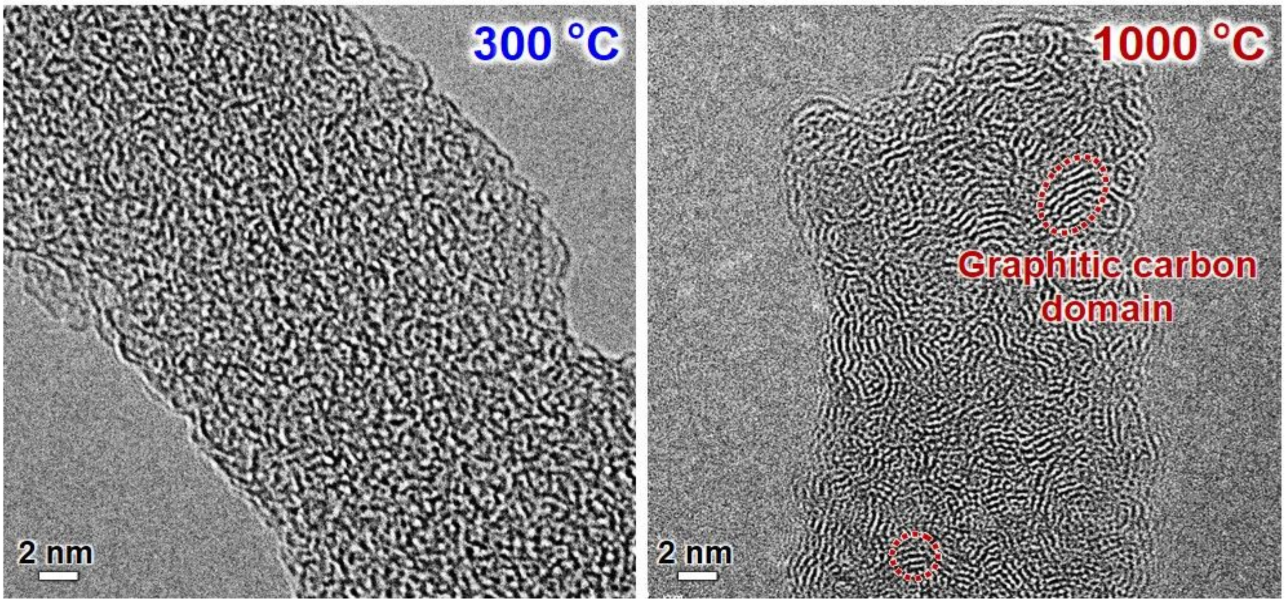

Figure S11. High-resolution transmission electron microscopy (HR-TEM) images of the cellulose nanofiber pyrolyzed at 300 and 1000 °C.

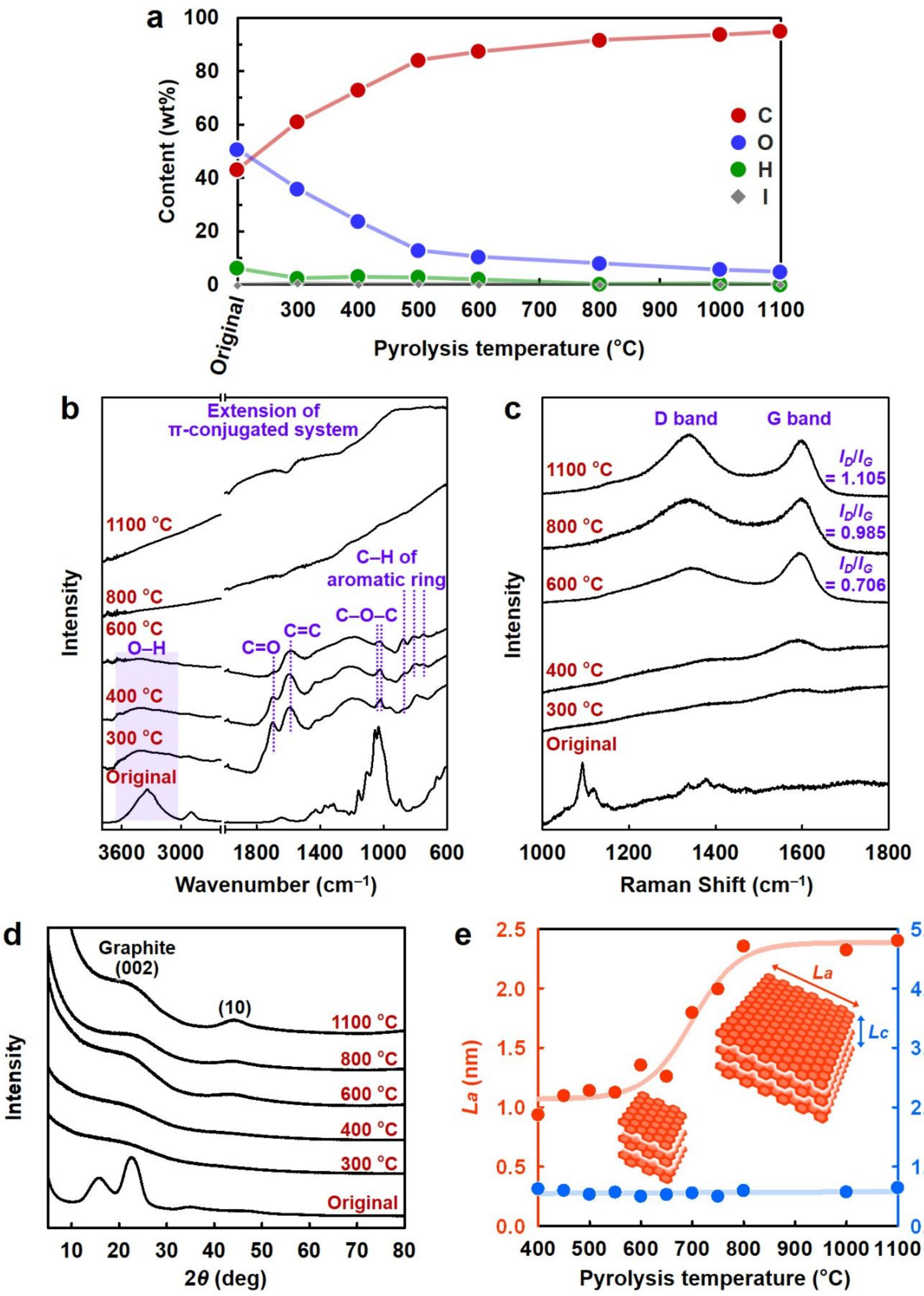

Figure S12. Molecular structures of pyrolyzed cellulose nanofiber. **(a)** Elemental content, **(b)** Fourier-transform infrared (FT-IR) spectra, **(c)** Raman spectra, **(d)** X-ray diffraction (XRD)

data, and **(e)** crystallite sizes of the graphene fragments in the in-plane (L_a) and stacking (L_c) directions for the cellulose nanofibers pyrolyzed at different temperatures.

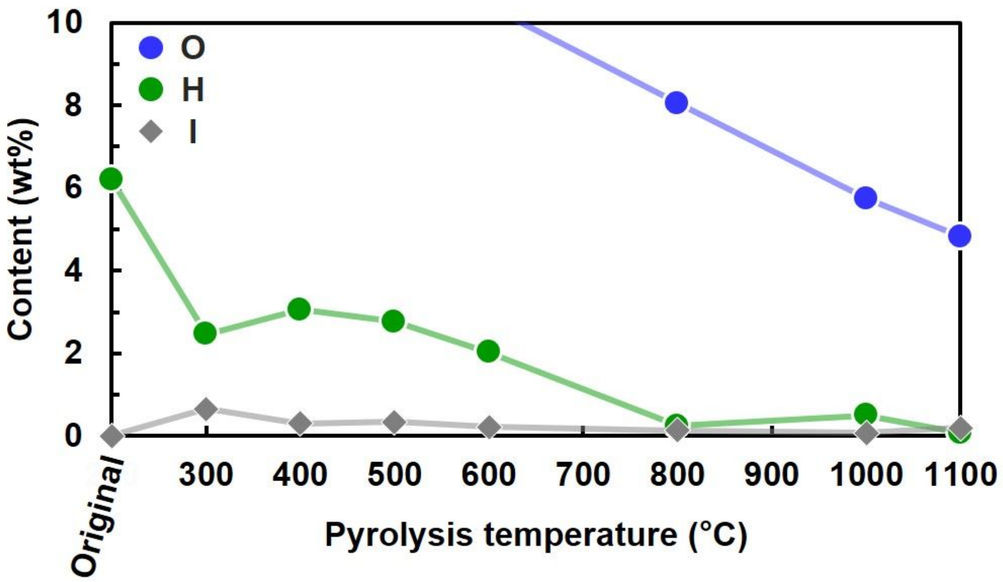

Figure S13. Elemental contents of the cellulose nanofiber pyrolyzed at different temperatures.

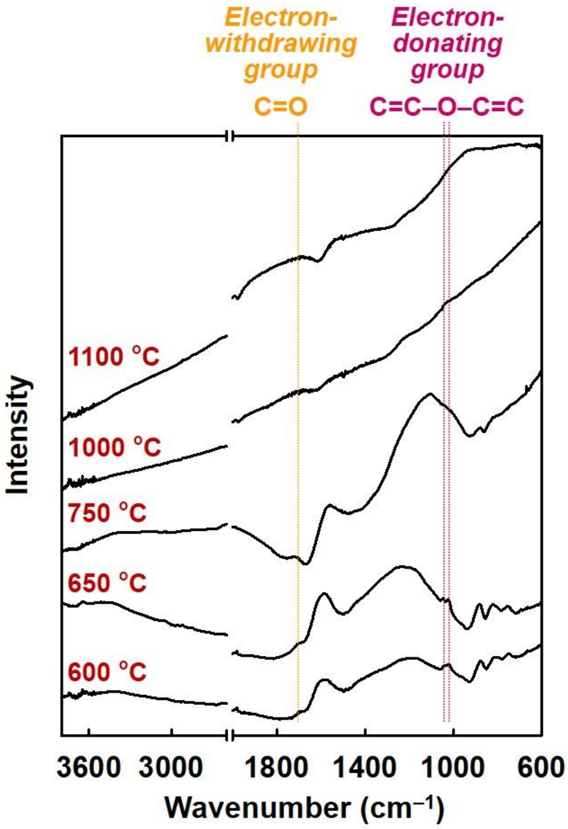

Figure S14. Fourier-transform infrared (FT-IR) spectra of the cellulose nanofiber pyrolyzed at different temperatures.

To explain the *n*-rich conduction property of the CNP pyrolyzed at 650 °C and the *p*-rich conduction properties of the CNPs pyrolyzed at 750, 1000, and 1100 °C, their functional groups were compared. The data for the CNP pyrolyzed at 650 °C show the presence of C=C–O–C=C group (furan-like ethers) with peaks at ~1060 and ~1010 cm^{-1},[20] and peaks corresponding to the C=O group at ~1700 cm^{-1}. As reported for reduced graphene oxides,[21] C=C–O–C=C acts as an electron-donating group and leads to *n*-type conduction, while C=O acts as an electron-withdrawing group and leads to *p*-type conduction. Therefore, for the CNP pyrolyzed at 650 °C, the electron-donating property exhibited by the C=C–O–C=C group would be predominant and result in *n*-rich conduction property. The CNP pyrolyzed at 600 °C may also exhibit *n*-rich conduction property, as it shows similar peaks as those of the CNP pyrolyzed at 650 °C. For the CNPs pyrolyzed at 750, 1000, and 1100 °C, the C=C–O–C=C

peak disappears, suggesting that the electron-withdrawing phenomenon exhibited by the C=O group may be predominant and lead to *p*-rich conduction property.

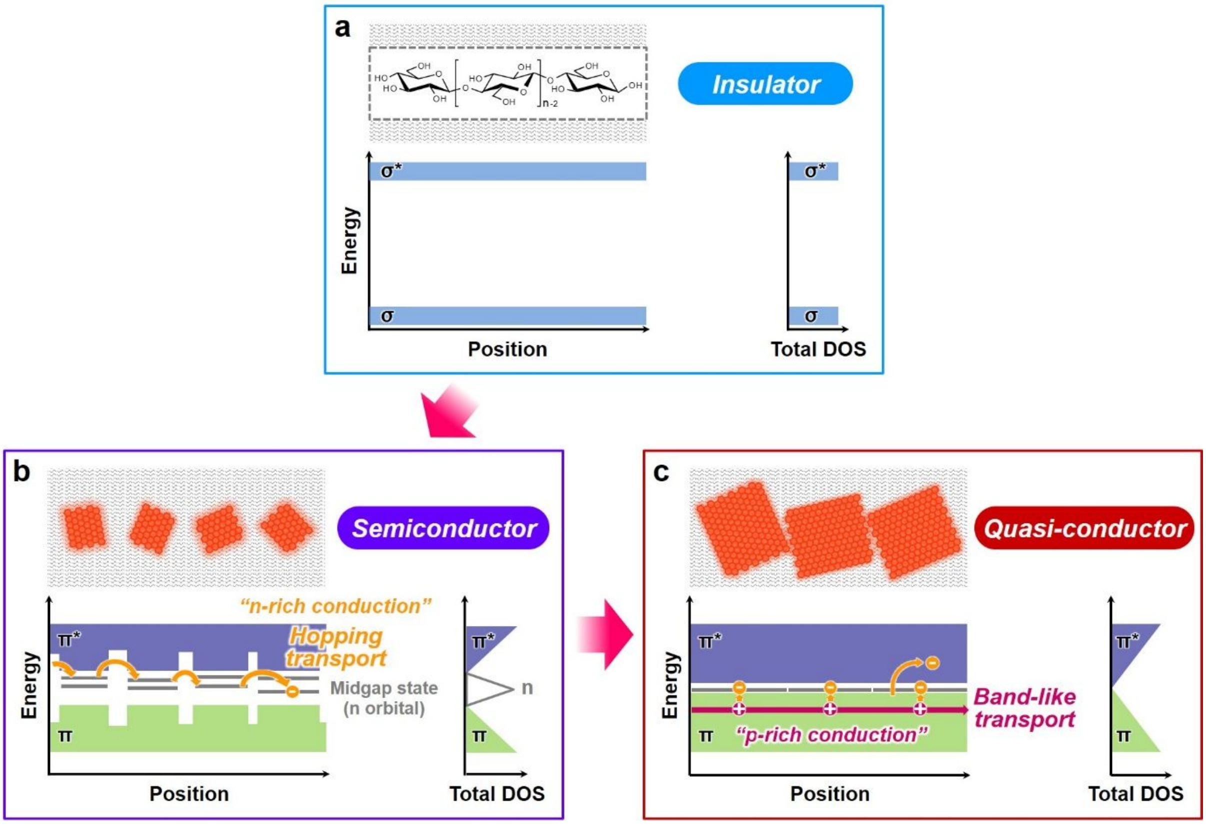

Figure S15. Putative mechanism for the drastic and progressive modulation of the electrical properties of cellulose nanofibers upon pyrolysis (DOS, density of states).

The putative mechanism for the drastic and progressive modulation of the electrical properties of pyrolyzed CNP is proposed. Original CNP is an electrical insulator with high electrical resistivity owing to the wide σ–σ^* energy gap of the sp^3-hybridized carbons (**Figure S15a**). The electrical resistivity modulation of the CNP is governed by the gradual growth of the sp^2-hybridized carbon fragments in the nanofibers upon pyrolysis. At low pyrolysis temperatures of ≤650 °C, pyrolyzed CNP has small graphitic sp^2-hybridized carbon domains (π-orbital) intermingled with disordered regions including oxygen-containing groups (n-orbital), in which the π–π^* energy gap and *n* energy level lie within the σ–σ* and π–π^* energy gaps, respectively.[22,23] Based on the result that the carrier transport activation energy of pyrolyzed CNP is lower than its bandgap, particularly in the low pyrolysis temperature range (**Figure 2c**

and **S6**), it is suggested that the carriers (*n*-rich) can pass through midgap states (n-orbital) within the π–π^* energy gap via hopping transport (**Figure S15b**). According to the *n*-rich conduction property, which is experimentally estimated, the midgap states should be located predominantly near the conduction band. At high pyrolysis temperatures of >650 °C, the electrical resistivity of pyrolyzed CNP can be further decreased by the growth of sp^2-hybridized carbon fragments because the π–π^* energy gap decreases with the growth of these domains.[22] The electronic structure and carrier transport mechanism of pyrolyzed CNP are considered to be similar to those of graphene oxide.[24] However, pyrolyzed CNP allows the ultrawide-range modulation of its electrical resistivity that is difficult to achieved with graphene oxide (**Table S1**), owing to the highly insulating nature of the original cellulose nanofibers. The electronic structure of pyrolyzed CNP also provides the possibility of carrier density modulation through the midgap states. When the sp^2-hybridized carbon fragments were further grown at higher pyrolysis temperatures, both the carrier transport activation energy and optical bandgap were almost zero (**Figure 2c** and **S6**), suggesting that the carriers (*p*-rich) enabled a quasi-band-like transport by decreasing the π–π^* energy gap with further growth of the sp^2-hybridized carbon domains (**Figure S15c**). Based on the *p*-rich conduction property, which was experimentally estimated, the midgap states would be located predominantly near the valence band. Although there are some previous reports describing the pyrolyzed cellulose nanofiber conductor,[25–27] this study provides new insights into the cellulose nanofiber semiconductor with widely and systematically tunable electrical properties as well as trans-scale structural designability.

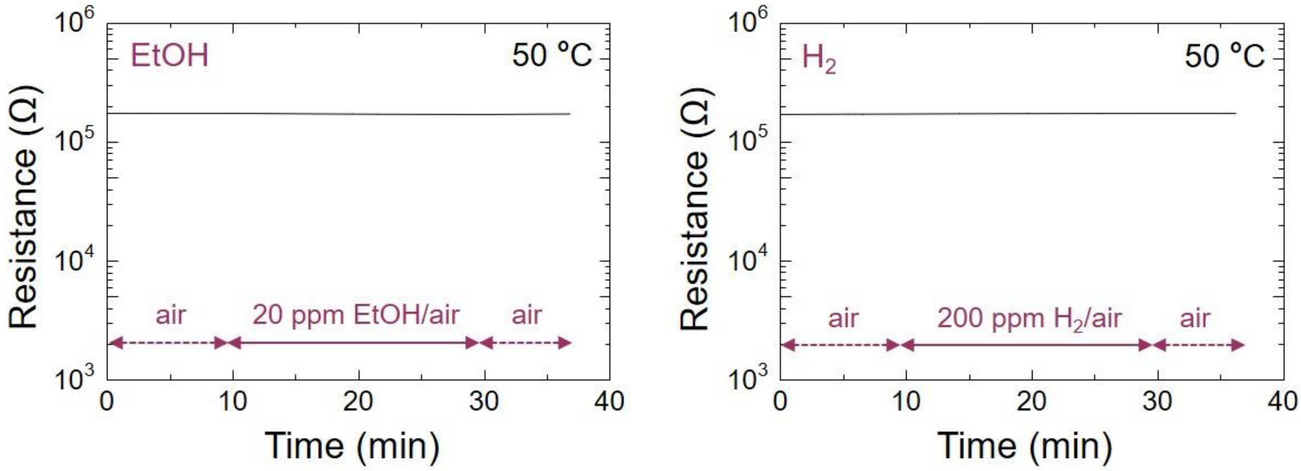

Figure S16. Water-vapor-selective sensing performance of the cellulose nanofiber paper (CNP) pyrolyzed at 600 °C. Resistance as a function of time upon exposure to ethanol (EtOH, 20 ppm) and H_2 (200 ppm) at 50 °C.

Pyrolyzed CNP shows almost no resistance change against EtOH (20 ppm) and H_2 (200 ppm) at 50 °C, suggesting that combustion reaction by flammable gases does not occur.

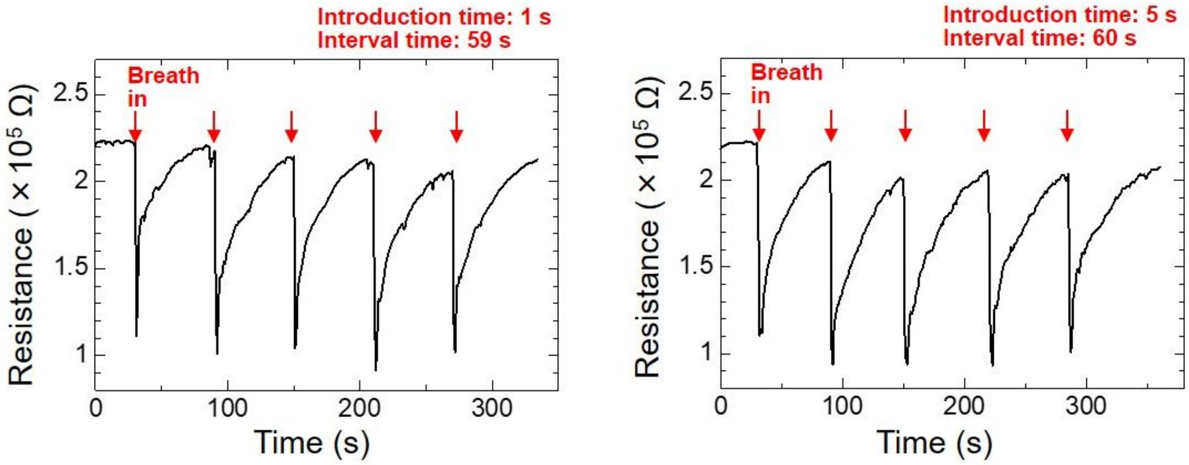

Figure S17. Water vapor sensing in human-exhaled breath using the cellulose nanofiber paper (CNP) pyrolyzed at 600 °C. Introduction time: 1 s (left) and 5 s (right).

There is no significant difference in resistance change upon exposure to human-exhaled breath within 5 s, regardless of the breath introduction time. Thus, one-second breathing is sufficient for water vapor sensing, indicating the fast response of the pyrolyzed CNP sensor.

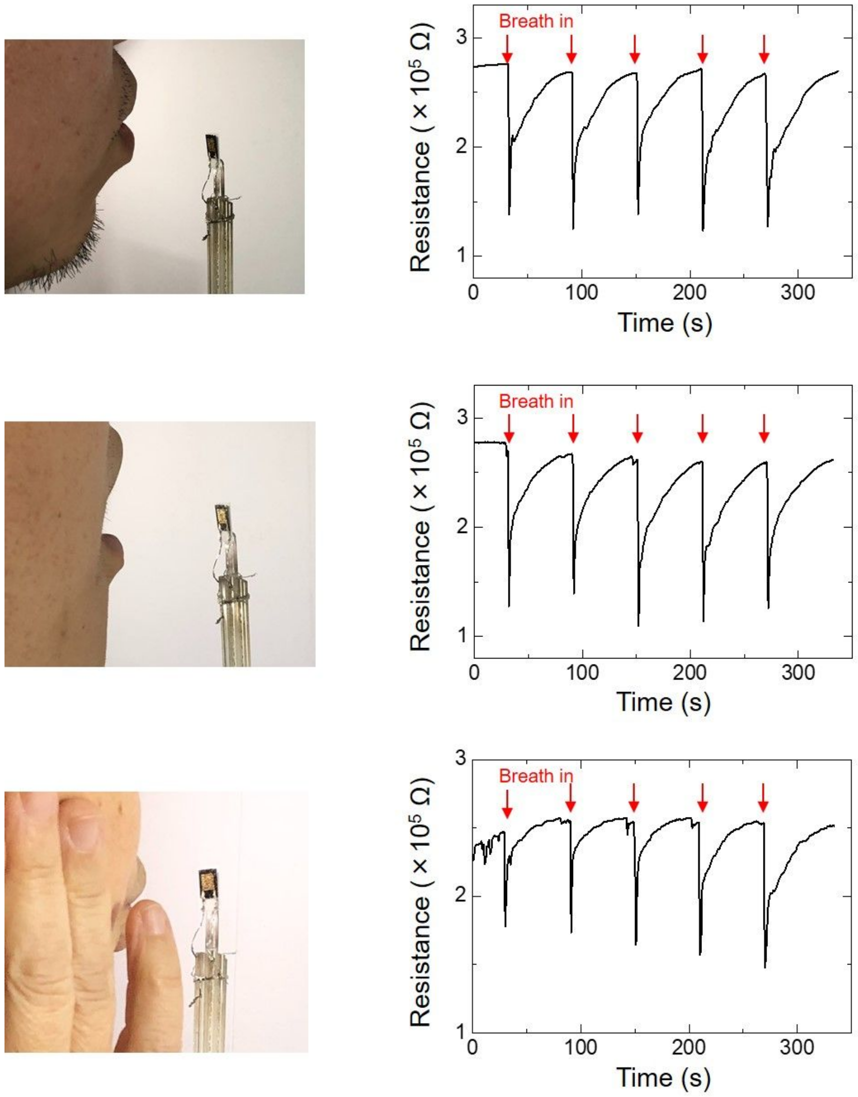

Figure S18. Water vapor sensing in human-exhaled breath using the cellulose nanofiber paper (CNP) pyrolyzed at 600 °C. Introduction time: 1s. Recovery time: 59 s.

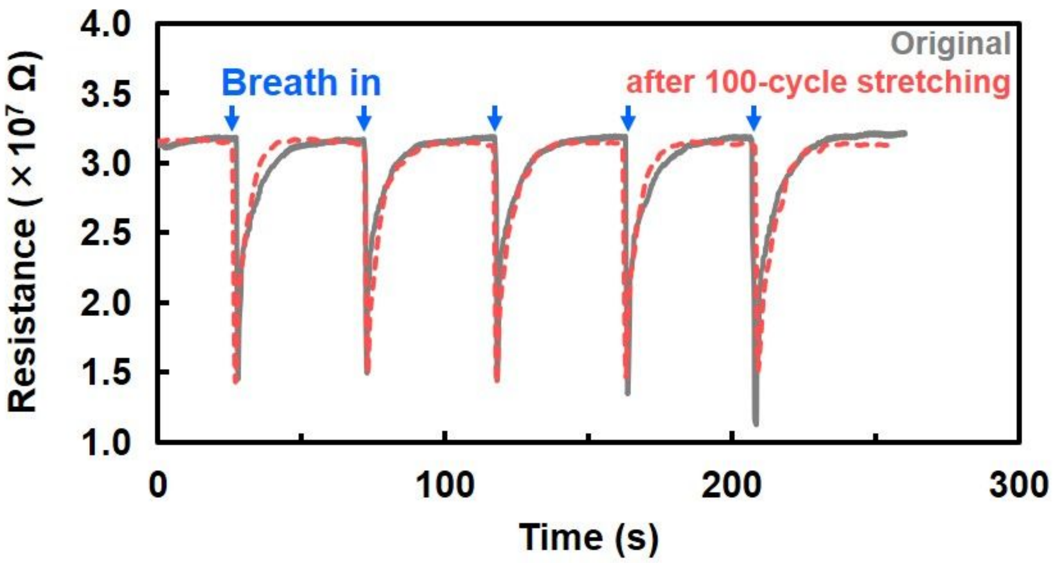

Figure S19. Water vapor sensing in human-exhaled breath by a sensor device that integrated the pyrolyzed cellulose nanofiber paper (CNP) sensor and *kirigami* paper substrate before (gray) and after (pink) 100-cycle of 160% stretching.

The device (**Figure 5a**) maintained its sensing performance even after 100-cycle stretching.

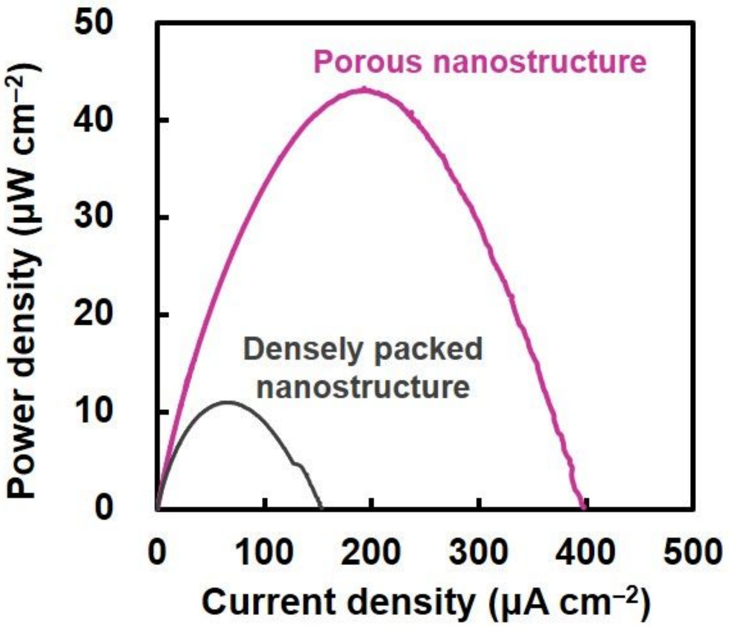

Figure S20. Biocatalytic electrode performance of the cellulose nanofiber paper (CNP) pyrolyzed at 800 °C for an enzymatic glucose biofuel cell. Power density as a function of current density for pyrolyzed CNPs with porous and densely packed nanostructures.

CNPs with porous and densely packed nanostructures were prepared with and without *t*-BuOH treatment, respectively, followed by pyrolysis under identical conditions (800 °C). The CNP with porous nanostructures pyrolyzed at 800 °C (specific surface area: ~643 m^2 g^{-1}) afforded a significantly higher power density than that observed with the densely packed nanostructures (specific surface area: ~80.5 m^2 g^{-1}). These results suggested that porous nanostructures increased the power density of pyrolyzed CNP, possibly by acting as an efficient immobilization field for the flavin adenine dinucleotide-dependent glucose dehydrogenase (FAD-GDH) enzyme (**Figure S21**).

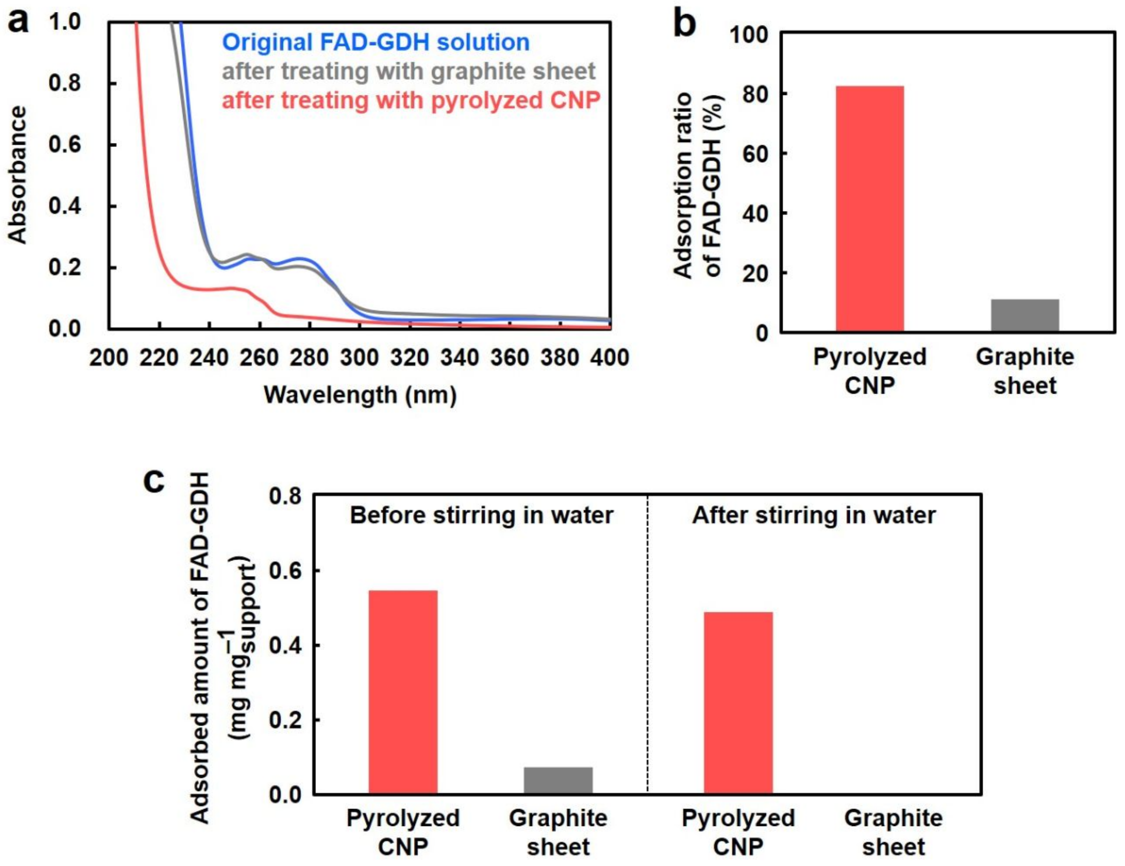

Figure S21. Flavin adenine dinucleotide-dependent glucose dehydrogenase (FAD-GDH) immobilization performances of the cellulose nanofiber paper (CNP) pyrolyzed at 1100 °C and graphite sheet.

For the evaluation of FAD-GDH immobilization behavior, 5 mg of the CNP pyrolyzed at 1100 °C with porous structures of pore sizes of ~100 nm (**Figure 1d**) or a commercial graphite sheet with a flat surface structure was first treated with the aqueous solution (MilliQ water) of FAD-GDH (3.3 mg mL^{-1}, 1 mL) for 20 h, followed by filtration. The filtrate was then subjected to UV–vis analysis. The adsorption ratio of FAD-GDH onto pyrolyzed CNP or graphite sheet was estimated by comparing the absorbance of the filtrate at 276 nm to that of the original FAD-GDH solution (**Figure S21a**). Pyrolyzed CNP showed a significantly higher adsorption ratio than the graphite sheet (**Figure S21b**), indicating that the porous fiber-network nanostructures with a high surface area of ~721 m^2 g^{-1} (**Figure S3**) of pyrolyzed

CNP contributed to the efficient adsorption of FAD-GDH. Furthermore, the adsorbed FAD-GDH on the pyrolyzed CNP support (~0.54 mg $mg_{support}^{-1}$) was efficiently retained even after stirring in water for 2 h (~0.49 mg $mg_{support}^{-1}$), while that on the graphite sheet support (~0.07 mg $mg_{support}^{-1}$) underwent desorption (**Figure S21c**). Thus, the retention ratios of the FAD-GDH adsorbed on the CNP pyrolyzed at 1100 °C and commercial graphite sheet were ~90% and ~0%, respectively (**Figure 6d**), suggesting that pyrolyzed CNP allowed effective FAD-GDH immobilization possibly owing to the porous fiber-network nanostructures.

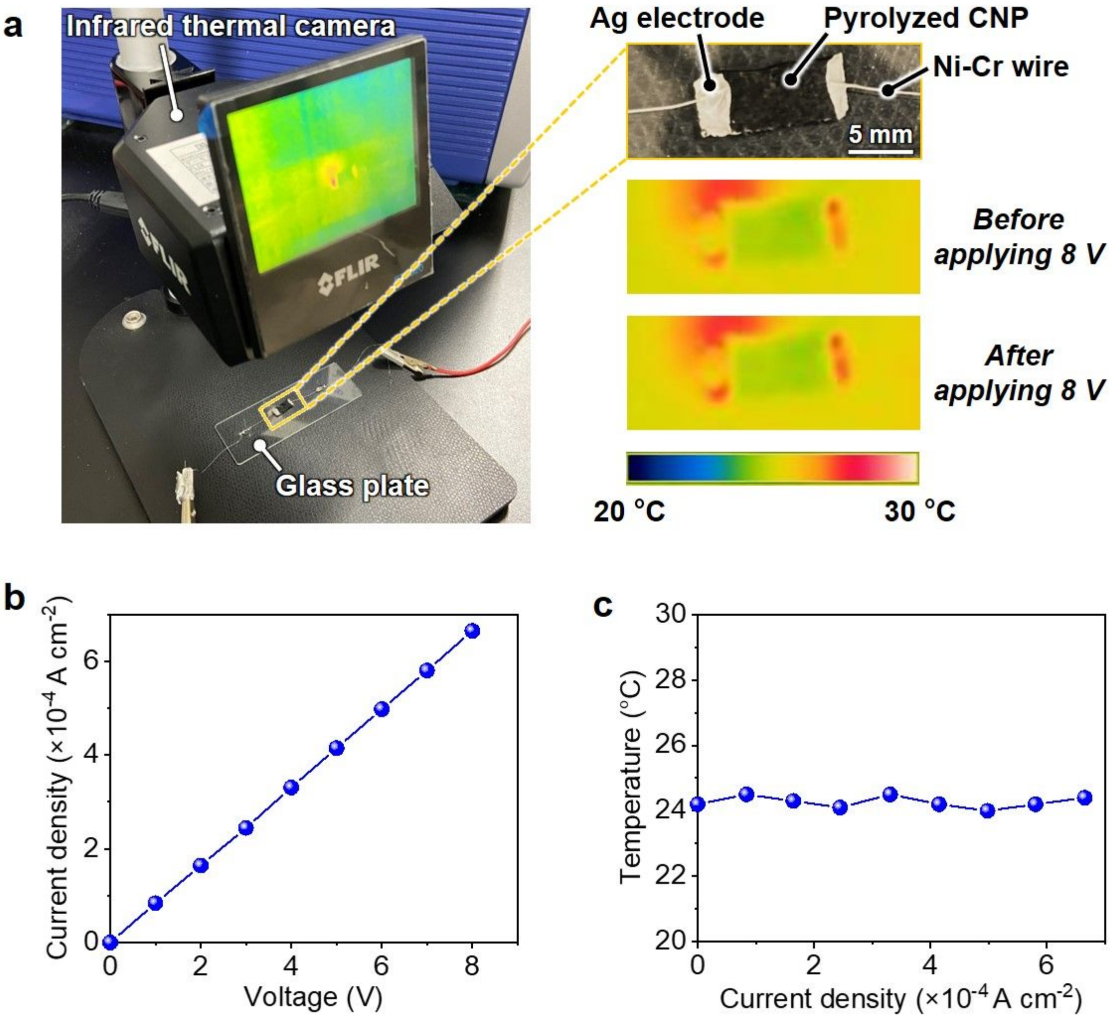

Figure S22. Measurements of current density and temperature for the cellulose nanofiber paper (CNP) pyrolyzed at 650 °C during applying voltage. (a) Experiment setup for surface temperature measurement during applying voltage, (b) current density versus voltage and (c) temperature versus current density. Distance between electrodes: ca. 8 mm, width of electrodes: ca. 5 mm, thickness of pyrolyzed CNP: 185 µm.

The carrier reversal or de-activation possibly occurs when the temperature of pyrolyzed CNP rises to the pyrolysis temperature that induces the *p*-*n* conversion (i.e. 650 °C) due to the Joule heating generated during the device operation. To investigate this possibility, the temperature of the CNP pyrolyzed at 650 °C was measured by an infrared thermal camera (FLIR ETS320, FLIR Systems. Inc., Wilsonville, USA) during applying voltage and current density up to 8 V

and 6.7 × 10^{-4} A cm^{-2}, respectively (**Figure S22a and b**). The results showed that the temperature of pyrolyzed CNP remained almost unchanged (**Figure S22c**), implying that the carrier reversal or de-activation would hardly occur at least in device applications presented in this study (applied voltage: 1 V).

Supporting Note 1 | Molecular structures of pyrolyzed cellulose nanofiber paper (CNP)

As the electrical insulating property of original CNP is derived from the sp^3-hybridized carbons present in the cellulose molecule,[28] the observed systematic variation of electrical properties in pyrolyzed CNPs must be associated with a progressive change in their molecular structures. To understand the change in the CNP molecular structure upon pyrolysis, the chemical and microscopic structures of pyrolyzed CNP were analyzed by elemental analysis, Fourier-transform infrared spectroscopy (FT-IR), NMR, Raman spectroscopy, X-ray diffraction (XRD) analysis, and transmission electron microscopy (TEM). Elemental analysis results showed that the carbon content in CNP gradually increased with an increase in the pyrolysis temperature and reached up to ~95 wt% at 1100 °C owing to the removal of oxygen and hydrogen (**Figure S12a**). The increase in the carbon content was more notably observed at temperatures of ≤600 °C. Additionally, only a small amount of iodine was detected at pyrolysis temperatures of >300 °C, indicating that effect of residual I_2 on the electrical properties of pyrolyzed CNP was almost negligible (**Figure S13**). The FT-IR spectra showed the formation of sp^2-hybridized carbon (C=C) and oxygen-containing groups (e.g., C=O, C–O–C, and C-OH) at low pyrolysis temperatures of ≤600 °C (**Figure S12b**). The intensities of peaks corresponding to the C=O, C–O–C, and C–OH functional groups decreased with an increase in the pyrolysis temperature. At high pyrolysis temperatures of ≥800 °C, the C=C peak disappeared, suggesting the extension of the π-conjugated system because of the growth of sp^2-hybridized carbon domains upon further pyrolysis.[29] These trends were consistently observed in the solid-state ^{13}C-NMR (**Figure 3a**) and Raman spectroscopy (**Figure S12c**) data. Specifically, in the solid-state ^{13}C NMR spectra, the peaks associated with C=O and O–C=C bonds were more conspicuous at low pyrolysis temperatures of ≤600 °C, and the peak for sp^2-hybridized carbon at approximately 120 ppm was more prominent at high pyrolysis temperatures of ≥800 °C, indicating the formation of more graphite-like structures.[30] In the

Raman spectra, the G band associated with the graphitic sp^2-hybridized carbon domains tended to be significant with increasing pyrolysis temperature. Notably, the D band associated with the disordered graphitic carbon structures (e.g., edge of graphitic domains and in-plane imperfections)[31] also became significant with an increase in the pyrolysis temperature. Such a trend was clearly observed by the increase in the intensity ratio of D and G band peaks (I_D/I_G) and could be interpreted in terms of the concomitant formation of disordered edge regions with the growth of the graphitic sp^2-hybridized carbon domains during pyrolysis.[32]

To further evaluate the growth of graphitic sp^2-hybridized carbon domains upon pyrolysis, XRD data were collected (**Figure S12d**). Two broad peaks assigned to the (10) and (002) reflections of graphite were observed at approximately 44° (interplanar distance d_a = ~0.2 nm) and 22° (d_c = ~0.4 nm), respectively, and these tended to be significant with increasing pyrolysis temperatures. The crystallite size of graphite in the in-plane (L_a) and stacking (L_c) directions was estimated using Scherrer's equation (**Figure S12e**).[33] With an increase in the pyrolysis temperature, L_a slightly increased from ~1.0 nm in the low-temperature range and rapidly increased to ~2.4 nm above 800 °C. In contrast, L_c remained constant at ~0.6 nm in the examined temperature range. These results suggested that the graphitic carbon domains progressively grew in the in-plane direction rather than in the stacking direction. The progressive growth of the graphitic carbon domains was also confirmed by the high-resolution TEM images shown in **Figure 3b** (see also **Figure S11**). Consequently, the graphitic carbon domains became nearly percolative at 1100 °C.

According to the above discussion, the dependence of the electrical properties of pyrolyzed CNP on pyrolysis temperature can be explained as follows. At low pyrolysis temperatures, the sp^2-hybridized carbon fragments are formed with disordered regions including C=O, C–O–C,

and C–OH groups. The sp^2-hybridized carbon fragments are separated from each other, and carrier hopping between the carbon fragments may dominate carrier transport. When the pyrolysis temperature is increased to approximately 600 °C, the number of sp^2-hybridized carbon fragments increases, and the disordered regions of carbon fragments tend to decrease with the gradual removal of C=O, C–O–C, and C–OH groups. Thermal reduction decreases the density of the midgap state and enhances the carrier transport, similar to the conduction mechanism of reduced graphene oxide.[24] Both the number of sp^2-hybridized carbon fragments and chemical state of the disordered regions determine the electrical properties at this stage. In contrast, at high pyrolysis temperatures, the growth of the graphitic sp^2-hybridized carbon domains incorporating the disordered edge in the domains becomes significant and dominates the electrical properties. The major carrier type can be determined by the chemical state of the disordered regions; *n*- and *p*-rich conductions are derived from electron-donating groups such as C=C–O–C=C (furan-like ethers) and electron-withdrawing groups such as C=O, respectively (**Figure S14**). All the observed phenomena continuously and progressively occur during pyrolysis. Thus, the drastic and systematic change in the electrical properties of pyrolyzed CNP can be interpreted in terms of the variation of the molecular structure via progressive pyrolysis (**Figure S15**).

References

1. Kondo, T.; Kose, R.; Naito, H.; Kasai, W. Aqueous Counter Collision Using Paired Water Jets As a Novel Means of Preparing Bio-Nanofibers. *Carbohydr. Polym.* **2014**, *112*, 284–290.

2. Saito, T.; Isogai, A. TEMPO-Mediated Oxidation of Native Cellulose. The Effect of Oxidation Conditions on Chemical and Crystal Structures of the Water-Insoluble Fractions. *Biomacromolecules*, **2004**, *5*, 1983–1989.

3. Chen, Z.; Ren, W.; Gao, L.; Liu, B.; Pei, S.; Cheng, H. M. Three-Dimensional Flexible and Conductive Interconnected Graphene Networks Grown by Chemical Vapour Deposition. *Nat. Mater.* **2011**, *10*, 424–428.

4. Qiu, L.; Liu, J. Z.; Chang, S. L. Y.; Wu, Y.; Li, D. Biomimetic Superelastic Graphene-Based Cellular Monoliths. *Nat. Commun.* **2012**, *3*, 1241.

5. Barg, S.; Perez, F. M.; Ni, N.; do Vale Pereira, P. d. V.; Maher, R. C.; Garcia-Tuñon, E.; Eslava, S.; Agnoli, S.; Mattevi, C.; Saiz, E. Mesoscale Assembly of Chemically Modified Graphene into Complex Cellular Networks. *Nat. Commun.* **2014**, *5*, 4328.

6. Worsley, M. A.; Kucheyev, S. O.; Satcher, J. H. Jr.; Hamza, A. V.; Baumann, T. F. Mechanically Robust and Electrically Conductive Carbon Nanotube Foams. *Appl. Phys. Lett.* **2009**, *94*, 073115.

7. Talin, A. A.; Centrone, A.; Ford, A. C.; Foster, M. E.; Stavila, V.; Haney, P.; Kinney, R. A.; Szalai, V.; El Gabaly, F. E.; Yoon, H. P.; Léonard, F.; Allendorf, M. D. Tunable Electrical Conductivity in Metal-Organic Framework Thin-Film Devices. *Science* **2014**, *343*, 66–69.

8. Li, H.; Chang, J.; Li, S.; Guan, X.; Li, D.; Li, C.; Tang, L.; Xue, M.; Yan, Y.; Valtchev, V.; Qiu, S.; Fang, Q. Three-Dimensional Tetrathiafulvalene-Based Covalent Organic Frameworks for Tunable Electrical Conductivity. *J. Am. Chem. Soc.* **2019**, *141*, 13324–13329.

9. Byun, Y.; Xie, L. S.; Fritz, P.; Ashirov, T.; Dincă, M.; Coskun, A. A Three-Dimensional Porous Organic Semiconductor Based on Fully sp^2-Hybridized Graphitic Polymer. *Angew. Chem. Int. Ed. Engl.* **2020**, *59*, 15166–15170.

10. Wang, L.; Lu, M.; Wang, X.; Yu, Y.; Zhao, X.; Lv, P.; Song, H.; Zhang, X.; Luo, L.; Wu, C.; Zhang, Y.; Jie, J. Tuning the *p*-Type Conductivity of ZnSe Nanowires *via* Silver Doping for Rectifying and Photovoltaic Device Applications. *J. Mater. Chem. A* **2013**, *1*, 1148–1154.

11. Sze, S. M.; Ng, K. K., *Physics of Semiconductor Devices*; John Wiley & Sons: Hoboken, NJ, **2006**.

12. Zhang, Y.; Mori, T.; Ye, J.; Antonietti, M. Phosphorus-Doped Carbon Nitride Solid: Enhanced Electrical Conductivity and Photocurrent Generation. *J. Am. Chem. Soc.* **2010**, *132*, 6294–6295.

13. Zheng, Y.; Liu, J.; Liang, J.; Jaroniec, M.; Qiao, S. Z. Graphitic Carbon Nitride Materials: Controllable Synthesis and Applications in Fuel Cells and Photocatalysis. *Energy Environ. Sci.* **2012**, *5*, 6717–6731.

14. Morimoto, N.; Kubo, T.; Nishina, Y. Tailoring the Oxygen Content of Graphite and Reduced Graphene Oxide for Specific Applications. *Sci. Rep.* **2016**, *6*, 21715.

15. Pei, S.; Cheng, H.-M. The Reduction of Graphene Oxide. *Carbon* **2012**, *50*, 3210–3228.

16. Mathkar, A.; Tozier, D.; Cox, P.; Ong, P.; Galande, C.; Balakrishnan, K.; Leela Mohana Reddy, A. L. M.; Ajayan, P. M. Controlled, Stepwise Reduction and Band Gap Manipulation of Graphene Oxide. *J. Phys. Chem. Lett.* **2012**, *3*, 986–991.

17. Tsuchiya, T.; Terabe, K.; Aono, M. In Situ and Non-Volatile Bandgap Tuning of Multilayer Graphene Oxide in an All-Solid-State Electric Double-Layer Transistor. *Adv. Mater.* **2014**, *26*, 1087–1091.

18. ASTM F76 – 86. Standard Test Methods for Measuring Resistivity and Hall Coefficient and Determining Hall Mobility in Single-Crystal Semiconductors. *ASTM International*: West Conshohocken, PA, USA **2002**.

19. Van der Pauw, L. J. A Method of Measuring the Resistivity and Hall Coefficient on Lamellae of Arbitrary Shape. *Phil. Technol. Rev.* **1958**, *20*, 220–224.

20. Acik, M.; Lee, G.; Mattevi, C.; Pirkle, A.; Wallace, R. M.; Chhowalla, M.; Cho, K.; Chabal, Y. The Role of Oxygen during Thermal Reduction of Graphene Oxide Studied by Infrared Absorption Spectroscopy. *J. Phys. Chem. C* **2011**, *115*, 19761–19781.

21. Tu, N. D. K.; Choi, J.; Park, C. R.; Kim, H. Remarkable Conversion Between *n*- and *p*-Type Reduced Graphene Oxide on Varying the Thermal Annealing Temperature. *Chem. Mater.* **2015**, *27*, 7362–7369.

22. Mathioudakis, C.; Kopidakis, G.; Kelires, P. C.; Patsalas, P.; Gioti, M.; Logothetidis, S. Electronic and Optical Properties of a-C from Tight-Binding Molecular Dynamics Simulations. *Thin Solid Films* **2005**, *482*, 151–155.

23. Li, M.; Cushing, S. K.; Zhou, X.; Guo, S.; Wu, N. Fingerprinting Photoluminescence of Functional Groups in Graphene Oxide. *J. Mater. Chem.* **2012**, *22*, 23374–23379.

24. Negishi, R.; Akabori, M.; Ito, T.; Watanabe, Y.; Kobayashi, Y. Band-Like Transport in Highly Crystalline Graphene Films from Defective Graphene Oxides. *Sci. Rep.* **2016**, *6*, 28936.

25. Wu, Z. Y.; Liang, H. W.; Chen, L. F.; Hu, B. C.; Yu, S. H. Bacterial Cellulose: A Robust Platform for Design of Three Dimensional Carbon-Based Functional Nanomaterials. *Acc. Chem. Res.* **2016**, *49*, 96–105.

26. Li, S. C.; Hu, B. C.; Ding, Y. W.; Liang, H. W.; Li, C.; Yu, Z. Y.; Wu, Z. Y.; Chen, W. S.; Yu, S. H. Wood-Derived Ultrathin Carbon Nanofiber Aerogels. *Angew. Chem. Int. Ed. Engl.* **2018**, *57*, 7085–7090.

27. Yao, B.; Peng, H.; Zhang, H.; Kang, J.; Zhu, C.; Delgado, G.; Byrne, D.; Faulkner, S.; Freyman, M.; Lu, X.; Worsley, M. A.; Lu, J. Q.; Li, Y. Printing Porous Carbon Aerogels for Low Temperature Supercapacitors. *Nano Lett.* **2021**, *21*, 3731–3737.

28. Klemm, D.; Heublein, B.; Fink, H. P.; Bohn, A. Cellulose: Fascinating Biopolymer and Sustainable Raw Material. *Angew. Chem. Int. Ed. Engl.* **2005**, *44*, 3358–3393.

29. Ning, J.; Hao, L.; Jin, M.; Qiu, X.; Shen, Y.; Liang, J.; Zhang, X.; Wang, B.; Li, X.; Zhi, L. A Facile Reduction Method for Roll-to-Roll Production of High Performance Graphene-Based Transparent Conductive Films. *Adv. Mater.* **2017**, *29*, 1605028.

30. Stankovich, S.; Dikin, D. A.; Piner, R. D.; Kohlhaas, K. A.; Kleinhammes, A.; Jia, Y.; Wu, Y.; Nguyen, S. T.; Ruoff, R. S. Synthesis of Graphene-Based Nanosheets *via* Chemical Reduction of Exfoliated Graphite Oxide. *Carbon* **2007**, *45*, 1558–1565.

31. Dresselhaus, M. S.; Jorio, A.; Souza Filho, A. G.; Saito, R. Defect Characterization in Graphene and Carbon Nanotubes Using Raman Spectroscopy. *Philos. Trans. A Math. Phys. Eng. Sci.* **2010**, *368*, 5355–5377.

32. Dey, R. S.; Hajra, S.; Sahu, R. K.; Raj, C. R.; Panigrahi, M. K. A Rapid Room Temperature Chemical Route for the Synthesis of Graphene: Metal-Mediated Reduction of Graphene Oxide. *Chem. Commun. (Camb)* **2012**, *48*, 1787–1789.

33. Biscoe, J.; Warren, B. E. An X-Ray Study of Carbon Black. *J. Appl. Phys.* **1942**, *13*, 364–371.
